# The Association Between Accelerated Biological Aging and the Physical, Psychological, and Cognitive Multimorbidity and Life Expectancy: Cohort Study

**DOI:** 10.1111/acel.70142

**Published:** 2025-07-13

**Authors:** Zuliyaer Talifu, Ziyang Ren, Chen Chen, Shuai Guo, Yu Wu, Yuling Li, Binbin Su, Xiaoying Zheng

**Affiliations:** ^1^ School of Population Medicine and Public Health Chinese Academy of Medical Sciences & Peking Union Medical College Beijing China; ^2^ Department of Epidemiology and Biostatistics, School of Public Health Peking University Beijing China; ^3^ Peking University Beijing China; ^4^ APEC Health Science Academy (HeSAy) Beijing China

**Keywords:** biological aging, chronic conditions, health outcomes, life expectancy, multimorbidity

## Abstract

As the global population ages, multimorbidity has become a critical public health issue. We analyzed 332,012 adults from the UK Biobank (2006–2022) to investigate the association between biological age—measured by the Klemera–Doubal method (KDM‐BA) and phenotypic age (PhenoAge)—and a new comorbidity model encompassing physical, psychological, and cognitive disorders, with overall mortality outcomes over a median follow‐up of 13.6 years. Logistic regression models examined the association between baseline health status and accelerated aging, while Cox proportional hazards models assessed mortality risk and disorder development. Cross‐sectional analysis showed that accelerated aging was linked to higher comorbidity prevalence. Longitudinal follow‐up revealed that individuals in the highest quartile (Q4) of aging speed (residual difference between estimated biological age and chronological age) had a 16%–17% higher risk of developing a single disorder, a 41%–44% higher risk of multimorbidity, and a 54% higher overall mortality risk compared with the lowest quartile (Q1). Among those with baseline single disorder, dual comorbidity, and triple morbidity, Q4 mortality risk increased by 89%–116%, 118%–166%, and 119%–156%, respectively. Multistate Markov models confirmed that accelerated aging (residual > 0) increased the risk of transitioning to disorder, comorbidity, and death by 12%–37%. Individuals aged 45 with triple comorbidity lost an average of 5.3 years in life expectancy (LE), further reduced by 5.8 to 7.0 years due to accelerated aging. This study highlights that KDM‐BA and PhenoAge robustly predict multimorbidity trajectories, mortality, and shortened LE, supporting their integration into risk stratification frameworks to optimize interventions for high‐risk populations.

## Graphical abstract

Accelerated biological aging (KDM‐BA/PhenoAge) predicts higher multimorbidity risks and 5.3–7.0 life‐years lost in 332,012 UK adults. Those with accelerated aging face 12%–37% increased transition risks to severe health states. Integrating these metrics enables targeted interventions to reduce chronic disease burdens in aging populations.

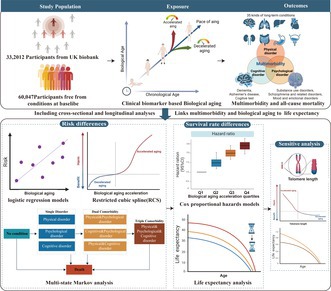

**Abbreviations**
BMI: body mass index
CGA: comprehensive geriatric assessment
CI: confidence interval
HR: hazard ratio
ICD‐10: International Classification of Diseases, 10th Revision
KMD‐BA: Klemera–Doubal method biological aging
MET: metabolic equivalent task
NHS: National Health Service
PhenoAge: phenotypic age
RCS: restricted cubic spline
SD: standard deviations
YLL: years of life lost

## 1 Introduction

The prevalence of multimorbidity on a global scale has become a significant public health concern (The Academy of Medical Science 2018). Multimorbidity is associated with adverse health outcomes, including decreased quality of life, higher healthcare costs, and an increased risk of premature death (Griffith et al. 2019; Vogeli et al. 2007). Notably, the accumulation of chronic conditions exacerbates mortality risk (Menotti et al. 2001), and each additional chronic condition is associated with an average reduction of 1.8 years in life expectancy (LE) (DuGoff et al. 2014). While COVID‐19 temporarily reversed decades of progress in LE, the long‐term burden of noncommunicable chronic disease (NCDs) and their comorbidities remain the dominant driver of mortality, particularly in aging populations (GBD 2021 Causes of Death Collaborators 2024). Socioeconomic disparities further compound this issue, as deprived communities experience multimorbidity onset up to a decade earlier than affluent groups, creating a synergistic “double burden” of accelerated biological aging and structural inequities (Skou et al. 2022). The coexistence of physical and mental disorders not only affects individual health statuses but may also lead to complex interactions among these conditions, exacerbating symptoms, and increasing the demand for healthcare services (Ni et al. 2023). Moreover, in this context, the early identification and proactive management of high‐risk individuals with multimorbidity, alongside the formulation of effective public health intervention strategies, presents significant challenges to achieving healthy aging.

Given the complexity of multimorbidity, the integrated management of multimorbidity is a key priority for age‐related public health issues (Zhou et al. 2023). A significant challenge in this context is the varying health statuses of individuals due to the heterogeneity of the aging process (Klemera and Doubal 2006; Yusri et al. 2024). Biological aging, particularly when accelerated, has garnered increasing attention for its potential role in the disruptions associated with the aging process (Ferrucci et al. 2020; Piening et al. 2020). These disruptions contribute significantly to the onset and progression of various diseases and encompass changes at the cellular and molecular levels that elevate the risk of developing multiple conditions (Duan et al. 2022; Jylhävä et al. 2017). Recent studies have indicated that accelerated aging is associated with a significant increase in the risk of rheumatoid arthritis (Chen et al. 2024), cardiometabolic multimorbidity (Jiang et al. 2024), chronic kidney disease (Zheng et al. 2024), anxiety and depression (Gao et al. 2023), and cancer (Mak et al. 2023). Furthermore, research has highlighted correlations between accelerated aging and life course‐related factors, such as prenatal smoking exposure (Cui et al. 2024), and childhood adversity (Yu et al. 2024). These findings suggest that accelerated aging can partially reflect an individual's cumulative exposure throughout life and is linked to a higher incidence of physical and mental disorders in later years. Consequently, understanding biological age and its implications for health may serve as a robust tool for predicting physical and mental health disorders and guiding preventive strategies aimed at improving the quality of life for aging populations.

To address these gaps, we leverage the UK Biobank cohort to investigate how accelerated biological aging influences the trajectory of multimorbidity encompassing physical, psychological, and cognitive disorders. Furthermore, we quantify the extent to which accelerated aging mediates the association between multimorbidity, overall mortality, and LE. Our study aims to quantify the association between accelerated aging and adverse health outcomes, thereby providing evidence to support the application of related biomarkers in population‐based settings.

## 2 Materials and Methods

### 2.1 Study Design and Participants

**FIGURE 1:**
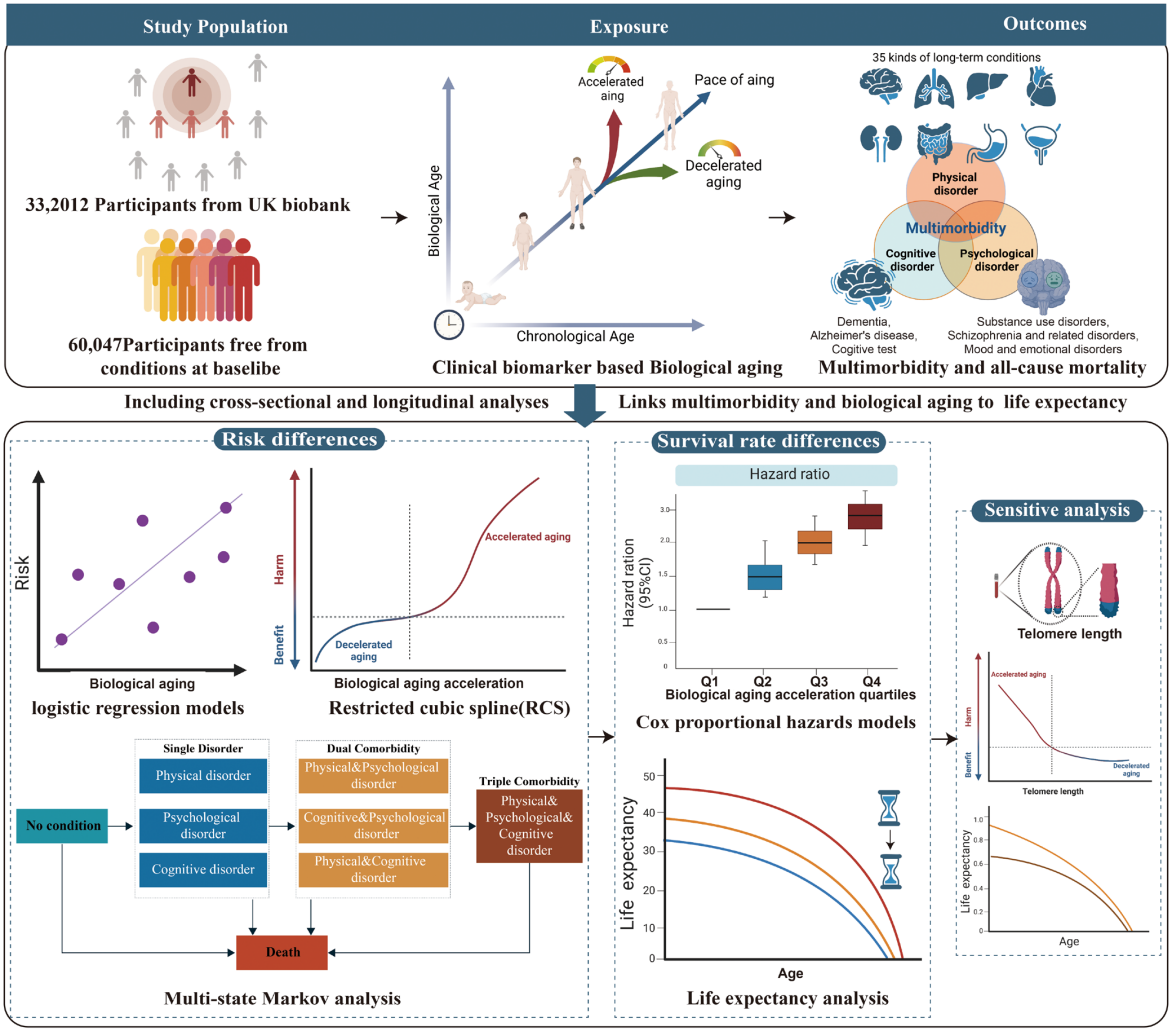
Flowchart of the overall study design.

The overall study design, as depicted in Figure 1, was based on data obtained from the UK Biobank (Application 105435). The UK Biobank comprises over 500,000 UK residents aged 37–73 years enrolled in this prospective cohort during 2006–2010, with comprehensive baseline health, lifestyle, and biospecimen data (Sudlow et al. 2015). In this study, after excluding participants lacking KDM‐BA/PhenoAge metrics (*n* = 170,056) or with extreme biological age values (those outside 5SD from mean, *n* = 112), 332,012 individuals retained analyzable data, as depicted in Figure S1. Further exclusion of 271,965 baseline prevalent cases resulted in 60,047 disorder‐free participants for evaluating associations between baseline biological age acceleration and incident multimorbidity/mortality.

### 2.2 Defining Multimorbidity

In our study, we established comorbidity patterns by incorporating specific long‐term physical, psychological, and cognitive conditions. We integrated disease records from multiple sources, including self‐reported diagnosed conditions, primary care records, inpatient diagnosis records, cancer registry, and death registry data, all categorized according to the International Classification of Diseases, 10th Revision (ICD‐10). Additionally, cognitive assessment data were incorporated into the analysis. For a detailed description of the data integration process and the specific sources utilized, please refer to Table S1. For physical disorders, we examined 35 long‐term conditions that may have enduring impacts on bodily health (Zhou et al. 2024), including cancer, chronic kidney disease, chronic obstructive pulmonary disease, diabetes, and liver disease. Psychological disorders were categorized as substance use disorder, schizophrenia, mood (affective) disorders, and neurotic, stress‐related, and somatoform disorders. During baseline assessment, cognitive disorder was defined to include Alzheimer's disease and other dementias, along with cognitive testing, which comprises assessments of fluid intelligence, reaction time, numeric memory, visuospatial memory, and prospective memory (Table S2). Throughout follow‐up, a diagnosis of Alzheimer's disease or other dementia was considered indicative of cognitive disorder. The last recorded date for disease information was October 31, 2022.

The classification of whether a disorder was present at baseline or developed during follow‐up was based on the dates of disease recording and the baseline survey. Participants were classified as having a specific disorder type if they met either of the following criteria: presence of at least one ICD code corresponding to a physical, psychological, or cognitive disorder, or a positive assessment on a cognitive impairment scale indicating cognitive disorder. Based on these criteria, individuals with only one disorder type (physical, psychological, or cognitive) were categorized as having a single disorder; those with two different disorder types were categorized as having dual comorbidity; and individuals with all three disorder types—physical, psychological, and cognitive—were classified as having triple comorbidity.

### 2.3 Mortality

Death records were obtained from the National Health Service (NHS) Information Centre for England and Wales, and the NHS Central Register for Scotland. The last recorded date of death was December 19, 2022.

### 2.4 Biological Aging

Biological age was assessed via two indices, the Klemera–Doubal method biological aging (KMD‐BA) and phenotypic age (PhenoAge), which leverage specific clinical biomarkers, as detailed in Table S3.

#### 2.4.1 Klemera–Doubal Method Biological Age (KDM‐BA)

The KDM‐BA is a composite biomarker‐based aging metric developed by Klemera and Doubal, which integrates clinical parameters associated with age‐related physiological decline (Klemera and Doubal 2006). For our study, we selected nine biomarkers spanning cardiovascular, metabolic, inflammatory, and renal function domains, each of which has been linked to aging processes. These biomarkers include systolic blood pressure, forced expiratory volume in 1 s, total cholesterol, glycated hemoglobin, urea, C‐reactive protein, alkaline phosphatase, albumin, and creatinine (Table S3). The KDM‐BA was calculated using a weighted least‐squares regression model:

$$\mathrm{KDM}‐\mathrm{BA}=\frac{\sum_{i=1}^{n}\left(x_{i}-q_{i}\right)\frac{k_{i}}{s_{i}^{2}}+\frac{\mathrm{CA}}{s_{\mathrm{BA}}^{2}}}{\sum_{i=1}^{n}{\left(\frac{k_{i}}{s_{i}^{2}}\right)}^{2}+\frac{1}{s_{\mathrm{BA}}^{2}}}$$

where *x*_*i*_ represents the individual measurements for each biomarker; and where *k*_*i*_, *q*_*i*_, *s*_*i*_ are the intercept, slope, and root mean square error, respectively. These values were estimated from the regression analysis of chronological age for each biomarker for both males and females. The term *s*_BA_ denotes the square root of the variance in chronological age explained by the selected set of biomarkers.

#### 2.4.2 Phenotypic Age (PhenoAge)

PhenoAge estimates the biological age at which an individual's mortality risk matches the population average (Levine et al. 2018). The algorithm was trained on the NHANES III cohort, a nationally representative US population with linked mortality data, ensuring generalizability to diverse aging trajectories. We calculated PhenoAge using chronological age and nine biomarkers predictive of mortality risk (Table S3); the mortality risk was derived as:

$$\text{PhenoAge}=141.50225+\frac{\ln\left[-0.00553\times \ln\left(1-\text{mortality risk}\right)\right]}{0.090165}$$

where $\text{mortality risk}=1-e^{-e^{\mathrm{xb}}\left[\exp\left(120\times \gamma \right)-1\right]/\gamma },\gamma =0.0076927$, 

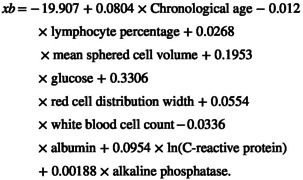




Then, “KDM‐BA acceleration” and “PhenoAge acceleration” were calculated as the residuals from regressing biological age on chronological age. Individuals with positive residuals were defined as having accelerated aging. All the data were derived from baseline blood and urine samples, along with relevant health metrics from routine physical examinations. Biological age indices were generated via the R package “Bioage” (Kwon and Belsky 2021). The construction of the biological age indices was informed by methodologies reported in previous studies (Cui et al. 2024).

### 2.5 Covariates

In this study, all covariates were assessed at baseline, including demographic, socioeconomic, lifestyle, and environmental factors that might influence the outcomes. The demographic variables included chronological age at recruitment, sex (male and female) and ethnicity (White and Others). Educational attainment was classified into levels: higher education, secondary education, professional and other qualifications, and no formal qualifications. To assess socioeconomic status, we employed the Townsend deprivation index alongside average total household income prior to tax, with the latter categorized into distinct income brackets.

Lifestyle factors included alcohol intake and smoking status (defined as never or ever), and body mass index (BMI) was divided into categories of normal weight (< 25 kg/m^2^), overweight (25–30), and obesity (> 30). Physical activity was measured in terms of metabolic equivalent task (MET) minutes per week, whereas dietary habits were assessed via a healthy diet score ranging from 0 to 5, with higher scores indicating a healthier dietary pattern (Table S4). Furthermore, air pollution variables included measurements of nitrogen dioxide (NO_2_), nitrogen oxides (NOx), fine particulate matter (PM2.5), and particulate matter with a diameter of 10 μm or less (PM10) in micrograms per cubic meter (μg/m^3^).

### 2.6 Statistical Analyses

All analyses were performed by using Stata 17.0 MP (*Stata* Corp.) and R version 4.3.0 (R Foundation for Statistical Computing). The baseline characteristics of the participants were reported on the basis of different comorbidity conditions. Continuous variables are presented as the means ± standard deviations (SDs), whereas categorical variables are reported as frequencies and percentages. To handle missing data, we implemented multiple imputation to generate 20 complete datasets. Ordered logistic regression models were used for imputing ordinal categorical variables, including household income and education level. For continuous variables potentially containing negative values (e.g., deprivation index), multivariate normal distribution imputation was applied, with values constrained to a plausible range. Non‐negative continuous variables, such as weekly physical activity levels and air pollution, were imputed using mean substitution. The distributions of the data before and after imputation were compared (see Figure S2). All analyses were conducted by pooling results from the 20 imputed datasets to ensure robustness and reliability of the findings.

#### 2.6.1 Cross‐Sectional Analyses

Logistic regression analysis was conducted to evaluate the associations between biological aging and the presence of single disorders and comorbidities at the baseline. The results are presented as odds ratios (ORs) with 95% confidence intervals (CIs), adjusted for potential confounding variables.

#### 2.6.2 Longitudinal Analyses

Among participants without pre‐existing disorders at baseline, we employed Cox proportional hazards models to examine longitudinal risks of incident disorder development, multimorbidity accumulation, and mortality. Hazard ratios (HRs) with 95% confidence intervals were calculated after controlling for key covariates. To characterize potential nonlinear relationships, restricted cubic spline (RSC) analyses were implemented to quantify the association gradient between biological aging acceleration and health outcomes.

A continuous‐time multistate Markov model was employed to estimate transition probabilities between distinct health states over the follow‐up period, including transient states (e.g., no condition, single disorder, and comorbidity) and an absorbing state (death). Transient states permit transitions to other health states or death, whereas the absorbing state (death) represents a terminal event with no further transitions (Figure S3). This approach, implemented in the R package “msm,” is well‐suited for analyzing health state transitions over time (Jackson 2011; Talifu et al. 2024). Transition intensities between states were modeled using proportional hazards, with baseline covariates incorporated as modifiers of transition rates. Parameters were estimated via maximum likelihood estimation, accounting for interval‐censored observation times.

Years of life lost (YLLs), which reflect the difference in life expectancy among different comorbidity patterns compared with a healthy state, were calculated by adapting a two‐step process from the literature (Chudasama et al. 2019; Lambert and Royston 2009). First, flexible parametric survival models were employed with age as the time scale to estimate residual life expectancy. This was done by calculating the area under the survival curve up to 100 years, conditional on survival from ages 45 to 100 in 1‐year intervals. Second, the differences in years of life were determined by comparing the areas under two survival curves, reflecting the life expectancy differences between comorbidity patterns and a healthy reference group. To determine life expectancy, proportional hazard survival analyses were performed via the stpm2 command, which applies restricted cubic splines to model the baseline cumulative hazard.

### 2.7 Sensitivity Analyses

To enhance robustness of our findings, the study incorporated two of the most commonly used biological age measures for analysis. Subsequently, we performed a series of sensitivity analyses to validate the results. First, we analyzed the dataset without imputation to evaluate the potential influence of covariate imputation on the outcomes. Next, we excluded individuals who experienced death or developed any disorder within the first 5 years of follow‐up to mitigate the risk of reverse causality. Finally, to further strengthen the analysis, we employed telomere length as an alternative biomarker of biological age (Jylhävä et al. 2017).

## 3 Results

### 3.1 Characteristics of Participants

Table 1 summarizes the baseline characteristics of these participants, categorized into no condition, single disorder, dual comorbidity, and triple comorbidity groups. The average age at recruitment was 56.4 years, with 54.0% of the participants being female. Biological age was greater among participants with multiple comorbidities than among those without corresponding conditions.

**TABLE 1:** Characteristics of study participants according to health status.

	Total	No condition	Single disorder^a^	Dual comorbidity^b^	Triple comorbidity^c^
	*N* = 332,012	*n* = 60,047	*n* = 183,505	*n* = 77,764	*n* = 10,696
Age at recruitment (years)	56.4 ± 8.1	53.3 ± 8.0	56.8 ± 8.0	57.7 ± 7.9	58.11 ± 7.73
Sex (female)	179,358 (54.02)	30,351 (50.55)	97,761 (53.27)	44,883 (57.72)	6363 (59.49)
Ethnicity (White)	313,754 (94.50)	57,481 (95.73)	175,520 (95.65)	71,019 (91.33)	9734 (91.01)
Missing	1512 (0.46)	196 (0.33)	711 (0.39)	503 (0.65)	102 (0.95)
Education levels					
College or university degree	107,587 (32.40)	24,359 (40.57)	61,281 (33.39)	19,951 (25.66)	1996 (18.66)
A/AS level or equivalent	37,375 (11.26)	7755 (12.91)	21,241 (11.58)	7585 (9.75)	794 (7.42)
O levels/GCSEs or equivalent	90,148 (27.15)	16,660 (27.74)	50,270 (27.39)	20,555 (26.43)	2663 (24.90)
Other (e.g., NVQ, nursing)	93,141 (28.05)	10,930 (18.20)	48,926 (26.66)	28,281 (36.37)	5004 (46.78)
Missing	3761 (1.13)	343 (0.57)	1787 (0.97)	1392 (1.79)	239 (2.23)
Townsend deprivation index	−1.37 ± 3.04	−1.65 ± 2.90	−1.54 ± 2.96	−0.93 ± 3.19	−0.18 ± 3.42
Missing	394 (1.2)	77 (1.3)	202 (1.1)	95 (1.2)	20 (1.9)
Average total household income before tax					
Less than 18,000	62,641 (18.87)	6330 (10.54)	31,781 (17.32)	20,713 (26.64)	3817 (35.69)
18,000 to 30,999	72,047 (21.70)	11,416 (19.01)	40,726 (22.19)	17,688 (22.75)	2217 (20.73)
31,000 to 51,999	74,830 (22.54)	15,749 (26.23)	42,810 (23.33)	14,779 (19.00)	1492 (13.95)
52,000 to 10,000	59,076 (17.79)	15,327 (25.53)	33,876 (18.46)	9154 (11.77)	719 (6.72)
Greater than 100,000	15,813 (4.76)	4665 (7.77)	9049 (4.93)	1967 (2.53)	135 (1.26)
Missing	47,626 (14.34)	6560 (10.92)	25,266 (13.77)	13,463 (17.31)	2316 (21.65)
Alcohol intake (never)	25,673 (7.73)	2838 (4.73)	12,698 (6.92)	8551 (11.00)	1586 (14.83)
Missing	655 (0.20)	39 (0.06)	211 (0.11)	326 (0.42)	79 (0.74)
Smoking status (never)	182,327 (54.92)	36,987 (61.60)	102,633 (55.93)	38,386 (49.36)	4321 (40.40)
Missing	1561 (0.47)	149 (0.25)	699 (0.38)	611 (0.79)	102 (0.95)
BMI (kg/m^2^)					
< 25	110,769 (33.43)	25,526 (42.53)	59,220 (32.33)	23,012 (29.70)	3011 (28.30)
25–30	141,777 (42.79)	25,547 (42.57)	79,115 (43.19)	32,689 (42.19)	4426 (41.60)
> 30	78,768 (23.77)	8945 (14.90)	44,841 (24.48)	21,779 (28.11)	3203 (30.10)
	698 (2.1)	29 (0.5)	329 (1.8)	284 (3.7)	56 (0.5)
Physical activity (MET, min/week)	2659.52 ± 2653.66	2633.77 ± 2592.72	2628.41 ± 2614.20	2726.73 ± 2768.25	2717.88 ± 2894.87
	74,131 (22.3)	10,661 (17.8)	39,529 (21.5)	20,545 (26.4)	3396 (31.8)
Healthy diet score					
0–1	113,463 (34.17)	22,250 (37.05)	62,812 (34.23)	25,060 (32.23)	3341 (31.24)
2–3	157,729 (47.51)	27,661 (46.07)	87,727 (47.81)	37,263 (47.92)	5078 (47.48)
4–5	33,322 (10.04)	5683 (9.46)	18,344 (10.00)	8226 (10.58)	1069 (9.99)
Missing	27,498 (8.28)	4453 (7.42)	14,622 (7.97)	7215 (9.28)	1208 (11.29)
Air pollution					
NO_2_ (μg/m^3^)	26.59 ± 7.58	26.48 ± 7.75	26.39 ± 7.55	27.01 ± 7.49	27.56 ± 7.47
Missing	4267 (1.3)	853 (1.4)	2317 (1.3)	957 (1.2)	140 (1.3)
NOx (μg/m^3^)	43.88 ± 15.50	43.50 ± 15.55	43.57 ± 15.43	44.64 ± 15.53	45.69 ± 15.91
Missing	4267 (1.3)	853 (1.4)	2317 (1.3)	957 (1.2)	140 (1.3)
PM2.5 (μg/m^3^)	9.98 ± 1.05	9.95 ± 1.07	9.96 ± 1.06	10.02 ± 1.04	10.11 ± 1.02
Missing	16,406 (4.9)	3327 (5.5)	9794 (5.5)	2974 (3.8)	311 (2.9)
PM10 (μg/m^3^)	16.22 ± 1.90	16.20 ± 1.92	16.20 ± 1.91	16.28 ± 1.87	16.34 ± 1.81
Missing	16,406 (4.9)	3327 (5.5)	9794 (5.5)	2974 (3.8)	311 (2.9)
Components of biological aging					
SBP (mmHg)	139.63 ± 19.56	135.73 ± 18.25	142.48 ± 19.98	145.28 ± 20.06	143.51 ± 19.67
Fev1 (L)	2.82 ± 0.80	2.95 ± 0.81	2.75 ± 0.78	2.58 ± 0.78	2.53 ± 0.75
Cholesterol (mmol/L)	220.67 ± 43.99	226.06 ± 41.36	217.40 ± 45.17	210.96 ± 46.81	209.74 ± 49.16
HBA1C (mmol/mol)	5.44 ± 0.60	5.32 ± 0.39	5.52 ± 0.67	5.66 ± 0.83	5.70 ± 0.86
Urea (mmol/L)	15.12 ± 3.84	14.76 ± 3.38	15.36 ± 4.09	15.67 ± 4.48	15.69 ± 4.59
C‐reactive protein (mg/L)	0.28 ± 0.46	0.24 ± 0.42	0.30 ± 0.48	0.35 ± 0.54	0.39 ± 0.60
Alkaline phosphatase (U/L)	83.22 ± 25.85	80.98 ± 25.08	84.44 ± 25.99	87.62 ± 27.34	89.19 ± 27.58
Albumin (g/L)	4.53 ± 0.26	4.53 ± 0.26	4.52 ± 0.26	4.51 ± 0.27	4.51 ± 0.28
Creatinine (μmol/L)	0.82 ± 0.20	0.81 ± 0.16	0.82 ± 0.22	0.83 ± 0.26	0.84 ± 0.37
Lymphocyte percentage (%)	28.99 ± 7.43	29.19 ± 7.23	28.85 ± 7.55	28.66 ± 7.74	28.46 ± 7.84
Mcv (fL)	82.80 ± 5.30	82.74 ± 5.19	82.79 ± 5.34	83.07 ± 5.56	83.48 ± 5.76
Glucose (mmol/L)	92.04 ± 21.57	88.75 ± 13.50	94.19 ± 25.19	97.51 ± 30.15	97.66 ± 28.94
Erythrocyte distribution width (%)	13.47 ± 0.96	13.43 ± 0.93	13.49 ± 0.98	13.58 ± 1.03	13.64 ± 1.02
Leukocyte count (10^9^ cells/L)	6.86 ± 1.92	6.68 ± 1.82	6.97 ± 2.01	7.18 ± 1.91	7.26 ± 1.99
Biological aging					
KDM‐BA	54.71 ± 8.99	50.60 ± 8.61	55.06 ± 8.81	56.66 ± 8.75	57.48 ± 8.56
KDM‐BA acceleration	−1.70 ± 4.14	−2.65 ± 3.80	−1.74 ± 4.12	−1.04 ± 4.25	−0.62 ± 4.38
PhenoAge	49.58 ± 9.74	45.18 ± 8.95	49.90 ± 9.54	51.75 ± 9.70	52.89 ± 9.67
PhenoAge acceleration	−6.83 ± 5.11	−8.06 ± 4.45	−6.90 ± 5.02	−5.95 ± 5.44	−5.22 ± 5.82
Telomere length	0.832 ± 0.131	0.844 ± 0.132	0.831 ± 0.130	0.826 ± 0.131	0.821 ± 0.127

Abbreviations: BMI, body mass index; Fev1, forced expiratory volume in 1 s; GCSE, general certificate of secondary education; HBA1C, glycated hemoglobin; KDM‐BA, Klemera‐Doubal biological age; MCV, mean sphered cell volume; MET, metabolic equivalent; NVQ, national vocational qualification; PhenoAge, phenotypic age; SBP, systolic blood pressure.

^a^ Single comorbidity, presence of one physical, psychological, or cognitive disorder.

^b^ Dual comorbidity, the presence of two disorders from any combination of physical, psychological, and cognitive.

^c^ Triple comorbidity, presence of all three types of disorders (physical, psychological, and cognitive).

At baseline, 60,047 individuals (18.1%) were classified as having no condition, were free from any of the included disorders, 166,254 individuals (50.1%) reported a single physical disorder, 5842 (1.8%) had a single psychological disorder, and 11,409 (3.4%) experienced a single cognitive disorder. Concurrent conditions included 36,121 participants (10.9%) with both physical and psychological disorders, 40,345 (12.1%) with both physical and cognitive disorders, and 1298 individuals (0.4%) who were found to have both psychological and cognitive disorders. Notably, 10,696 individuals (3.2%) presented with all three disorders. These baseline comorbidity patterns are illustrated in Figure S4A.

During a median follow‐up of 13.6 years, 26,886 participants (8.1%) died (Figure S4B). Among the 305,126 survivors, 52,934 individuals (15.9%) remained healthy with no conditions. A total of 133,057 participants (40.1%) only had physical disorder, 9930 (3.0%) only had psychological disorder, and 5508 (1.7%) only had cognitive disorder. Additionally, 54,668 participants (16.5%) had both physical and psychological disorders, 31,614 individuals (9.5%) had both physical and cognitive disorders, and 1219 individuals (0.4%) had both psychological and cognitive disorders. Triple comorbidities were present in 16,196 individuals (4.9%).

### 3.2 Accelerated Biological Aging and the Prevalence of Multimorbidity at the Baseline

For participants with a single disorder, KDM‐BA acceleration was associated with increased risk (OR: 1.03, 95% CI: 1.01–1.05, *p* < 0.001). While, PhenoAge acceleration did not significantly increase risk (OR: 1.01, 95% CI: 0.99–1.03, *p* = 0.205). When analyzing the data by quartiles, individuals in the fourth quartile of both KDM‐BA and PhenoAge exhibited significantly higher odds, with a 59% and 61% increase, respectively (OR: 1.59, 95% CI: 1.54–1.64; OR: 1.61, 95% CI: 1.56–1.66, both *p* < 0.001) (Table S5).

In the context of dual comorbidity, both KDM‐BA and PhenoAge acceleration were significantly associated with increased odds (KDM‐BA: OR: 1.07, 95% CI: 1.05–1.10, *p* < 0.001; PhenoAge: OR: 1.04, 95% CI: 1.01–1.06, *p* < 0.001). Notably, the quartile analysis revealed even more pronounced increases in odds. Specifically, individuals in the fourth quartile of KDM‐BA exhibited a 93% increase in odds (OR: 1.93, 95% CI: 1.86–2.00, *p* < 0.001), while those in the fourth quartile of PhenoAge showed a 107% increase in odds (OR: 2.07, 95% CI: 2.00–2.14, *p* < 0.001).

For triple comorbidity, each standard deviation (SD) increase in KDM‐BA acceleration was associated with a 20% increase in odds (OR: 1.20, 95% CI: 1.15–1.26, *p* < 0.001), while PhenoAge acceleration was associated with a 12% increase in odds (OR: 1.12, 95% CI: 1.07–1.17, *p* < 0.001). In the quartile analysis, individuals in the fourth quartile of KDM‐BA exhibited a 118% increase in odds (OR: 2.18, 95% CI: 2.03–2.34, *p* < 0.001), and those in the fourth quartile of PhenoAge showed an even greater increase of 169% in odds (OR: 2.69, 95% CI: 2.51–2.88, *p* < 0.001). Trend analyses across quartiles for both measures revealed significant linear trends for all conditions (*p* for trend < 0.001).

### 3.3 Association Between Biological Aging Acceleration and the Risk of Chronic Multimorbidity in Baseline Disorder‐Free Individuals

To investigate the associations between accelerated aging and different patterns of multimorbidity, we selected 60,047 individuals who were free of any diagnosed disorders at baseline. Among these participants, the number of individuals who developed somatic‐cognitive‐mental triple comorbidity during follow‐up was relatively low (*n* = 112). To ensure the robustness of the results, both dual and triple comorbidity were combined into a single group for analysis (Table S6).

**FIGURE 2:**
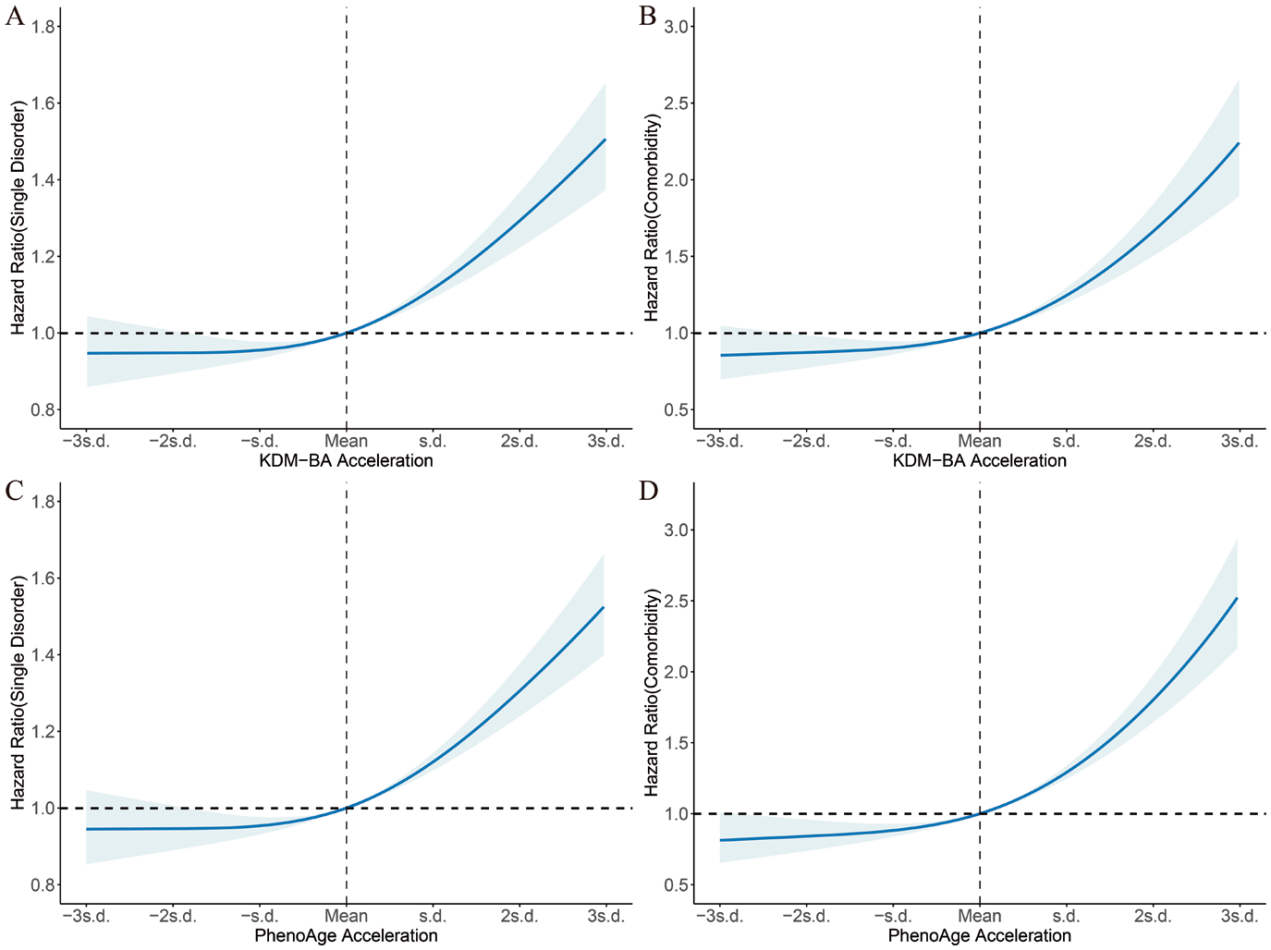
Dose–response relationships of KDM‐BA and PhenoAge accelerations with risks of single disorder and multimorbidity during follow‐up in baseline disorder‐free population. (A, B) Associations of KDM‐BA acceleration with the risk of (A) single disorder, (B) comorbidity. (C, D) associations of PhenoAge acceleration with the risk of (C) single disorder, (D) comorbidity. The models were adjusted for age, sex, ethnicity, BMI, smoking status, alcohol intake, healthy diet, physical activity, Townsend deprivation index, household income, and air pollution.

KDM‐BA acceleration was associated with a 12% higher risk of incident single disorder (HR: 1.12, 95% CI: 1.09–1.15, *p* < 0.001) and a 32% higher risk of incident dual/triple comorbidity (HR: 1.32, 95% CI: 1.23–1.41, *p* < 0.001). In the quartile analysis, individuals in the fourth quartile of KDM‐BA acceleration exhibited a 16% higher risk of incident single disorder (HR: 1.16, 95% CI: 1.13–1.20, *p* < 0.001) and a 44% higher hazard of incident dual/triple comorbidity (HR: 1.44, 95% CI: 1.32–1.57, *p* < 0.001). Similarly, PhenoAge acceleration was associated with a 19% higher risk of incident single disorder (HR: 1.19, 95% CI: 1.13–1.26, *p* < 0.001) and a 49% higher risk for dual/triple comorbidity (HR: 1.49, 95% CI: 1.32–1.68, *p* < 0.001). In the quartile analysis, individuals in the fourth quartile of PhenoAge acceleration showed a 17% higher risk of incident single disorder (HR: 1.17, 95% CI: 1.14–1.21, *p* < 0.001) and a 41% higher risk of incident dual/triple comorbidity (HR: 1.41, 95% CI: 1.30–1.54, *p* < 0.001). Dose–response analyses further demonstrated that both KDM‐BA and PhenoAge accelerations were significantly associated with an increased hazard of incident disorders (Figure 2).

### 3.4 Accelerated Aging and Mortality Risk Under Different Baseline Conditions

The results of the Cox proportional hazards model indicate that accelerated aging significantly increases the risk of mortality, with heightened risks observed in individuals with chronic conditions and comorbidities (Figure S5). For individuals without any conditions, those in the fourth quartile of KDM‐BA and PhenoAge accelerated aging had the same 54% increased risk of mortality compared with those in the first quartile (HR: 1.54, 95% CI: 1.37–1.173; HR: 1.54, 95% CI: 1.37–1.72, both *p* < 0.001). In individuals with a single disorder, the risk escalated to 89% (HR: 1.89, 95% CI: 1.80–1.198, *p* < 0.001) and 116% (HR: 2.16, 95% CI: 2.06–2.27, *p* < 0.001). For those with dual comorbidities, the risk further increased to 118% (HR: 2.18, 95% CI: 2.04–2.32, *p* < 0.001) and 166% (HR: 2.66, 95% CI: 2.47–2.86, *p* < 0.001). When experiencing triple comorbidities, the risk levels were comparable to those with dual comorbidities, with increases of 119% (HR: 2.19, 95% CI: 1.87–2.57, *p* < 0.001) and 156% (HR: 2.56, 95% CI: 2.14–3.06, *p* < 0.001). Additionally, the analysis of mortality risk associated with accelerated aging revealed a linear growth pattern for both KDM‐BA and PhenoAge, demonstrating that higher levels of accelerated aging were significantly associated with increased mortality risk across different baseline health status groups (Figure S6). Notably, this risk was more pronounced in individuals with multiple comorbidities at baseline.

### 3.5 Accelerated Aging and the Risk of Transitioning Between Different States

**FIGURE 3:**
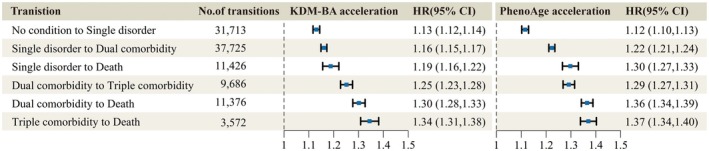
Hazard ratios (HRs) and 95% confidence intervals (CIs) for transition risks associated with KDM‐BA and PhenoAge accelerations. In the multistate Markov model, sex, age, and PhenoAge/KDM‐BA acceleration (converted to standard deviations) were included as covariates. Only 99 cases transitioned from “No condition” to death, and these cases were excluded from the analysis to ensure model stability.

The multistate Markov model analysis indicated that individuals with accelerated aging, whether assessed by KDM‐BA or PhenoAge, faced a significantly elevated risk of disorders, comorbidities, and mortality (Figure 3). Specifically, the transition from healthy to single disorder was associated with a 13% increase for accelerated KDM‐BA (HR: 1.13, 95% CI: 1.12–1.14) and a 12% increase in risk for accelerated PhenoAge (HR: 1.12, 95% CI: 1.10–1.13). Furthermore, the transition from single disorder to dual comorbidity indicated a risk increase of 16% for KDM‐BA (HR: 1.16, 95% CI: 1.15–1.17) and 22% for PhenoAge (HR: 1.22, 95% CI: 1.21–1.24). When assessing the transition from single disorder to death, risks increased by 19% for KDM‐BA (HR: 1.19, 95% CI: 1.15–1.17) and 30% for PhenoAge (HR: 1.30, 95% CI: 1.27–1.33). The risk of transitioning from dual comorbidity to triple comorbidity increased by 25% for KDM‐BA (HR: 1.25, 95% CI: 1.23–1.28) and 29% for PhenoAge (HR: 1.29, 95% CI: 1.27–1.29). Additionally, the transition from Dual Comorbidity to Death exhibited a 30% risk increase for KDM‐BA (HR: 1.30, 95% CI: 1.28–1.33) and a substantial 36% increase for PhenoAge (HR: 1.36, 95% CI: 1.34–1.39). Notably, among individuals with baseline triple comorbidities, accelerated KDM‐BA was associated with a 34% increase in mortality risk (HR: 1.34, 95% CI: 1.31–1.38), while accelerated PhenoAge corresponded to a 37% increase in mortality risk (HR: 1.37, 95% CI: 1.34–1.40).

### 3.6 Life Expectancy

#### 3.6.1 Multimorbidity

**FIGURE 4:**
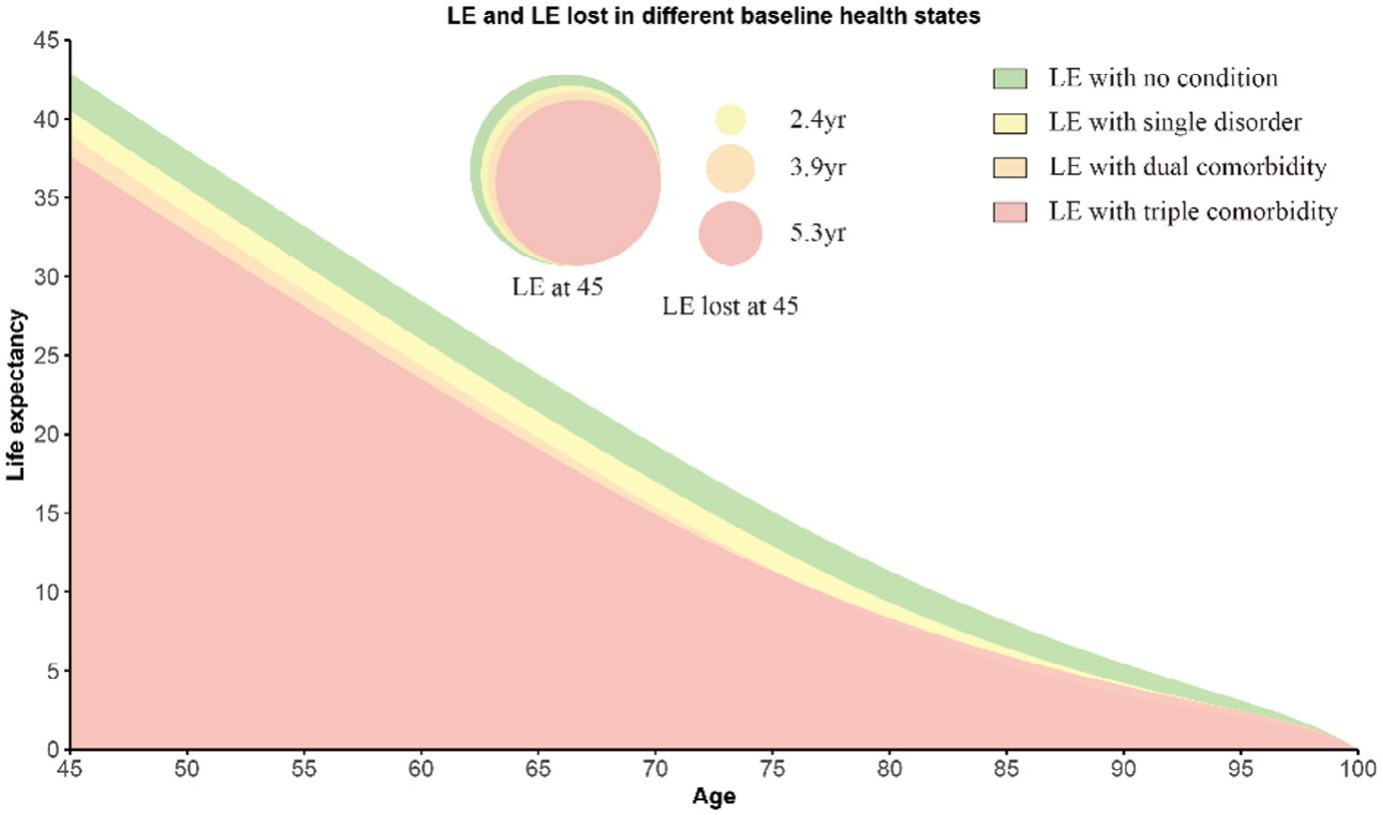
Life expectancy and years of life lost associated with single, dual, and triple disorders compared with healthy individuals across age groups. Models were adjusted for age, sex, ethnicity, BMI, smoking status, alcohol intake, healthy diet, physical activity, Townsend deprivation index, household income, and air pollution.

At Age 45, individuals without any disorders have an average life expectancy of 42.9 years (95% CI 41.1, 44.7), corresponding to a projected lifespan of approximately 87.9 years (Table S7). Those with a single disorder experienced a life expectancy of 40.5 years (95% CI 39.7, 41.3), reflecting a decline of 2.4 years. Life expectancy further decreases for individuals with dual disorders to 39.0 years (95% CI 38.0, 39.9), representing a reduction in 3.9 years. The most substantial decline was observed in individuals with triple disorders, who had an average life expectancy of 37.6 years (95% CI 35.5, 39.8), indicating a reduction in 5.3 years (Figure 4).

#### 3.6.2 Biological Aging Acceleration

On the basis of various baseline health conditions, individuals experiencing accelerated biological aging are expected to experience varying levels of life expectancy reduction (Figure S7). For those with no condition, at Age 45, individuals in the Q4 group of PhenoAge acceleration had a reduced LE by 2.9 years (95% CI: 2.0, 3.9) compared with those in the Q1 group, whereas those experiencing KDM‐BA acceleration had a reduction in 2.9 years (95% CI: 1.9, 3.9). For those with a single disorder, the Q4 group of PhenoAge acceleration had an LE reduction in 5.4 years (95% CI: 4.8, 6.0), while the KDM‐BA accelerated individuals had a reduction in 4.5 years (95% CI: 4.0, 5.0). In the presence of dual comorbidities, the Q4 group of PhenoAge acceleration experienced an LE reduction in 7.0 years (95% CI: 6.2, 7.8), whereas the KDM‐BA accelerated individuals experienced a reduction in 5.7 years (95% CI: 5.0, 6.4). For those with triple comorbidities, the reduction in LE for the Q4 group of PhenoAge acceleration was 6.9 years (95% CI: 4.9, 8.9), while the KDM‐BA acceleration group showed a reduction in 5.8 years (95% CI: 4.1, 7.7).

### 3.7 Sensitivity Analyses Results

First, we conducted an analysis using nonimputed data to evaluate the potential impact of covariate imputation on the results. The findings from this analysis were consistent with our primary results, demonstrating robustness in the observed associations (Tables S8 and S9). Second, in the analysis of the baseline disorder‐free population, we further excluded individuals who developed physical, psychological, or cognitive disorders or died within the first 5 years of follow‐up. This exclusion was implemented to minimize the possibility of reverse causality between adverse health outcomes and mortality (Figure S8). The results confirmed that accelerated biological aging, as measured by KDM‐BA and PhenoAge, remained a strong predictor of adverse health outcomes even in individuals without pre‐existing conditions (Table S10). Finally, we utilized telomere length as an alternative biomarker of biological age. The analysis revealed that shorter telomeres were associated with an increased risk of multimorbidity, higher mortality risk, and reduced life expectancy (Tables S11 and S12, Figures S9 and S10). Notably, the risk of reduced life expectancy associated with telomere shortening was lower than that associated with accelerated biological age.

## 4 Discussion

In this study, we tested the risk of comorbidities involving physical, mental, and cognitive disorders among more than 330,000 middle‐aged and older adults in the UK Biobank, via biological age, which was constructed from physiological and biochemical indicators. The key findings suggest that an accelerated biological age is significantly associated with a greater likelihood of comorbid physical, psychological, and cognitive disorders, as well as an increased risk of comorbidity and mortality over an average follow‐up period of 13.6 years. Moreover, the onset of such comorbidities significantly shortens an individual's life expectancy. Notably, individuals experiencing accelerated aging experience varying degrees of reduced life expectancy, depending on their baseline health status.

In our study, two commonly used biological age metrics were utilized. The PhenoAge model provided more stable risk predictions for multimorbidity than the KDM‐BA model, likely due to the lower proportion of individuals identified as having accelerated aging by PhenoAge (28,233 individuals, 8.7% of the baseline population) compared with the KDM‐BA model (105,509 individuals, 31.6% of the baseline population), as outlined in Table S5. The smaller subset of individuals classified as accelerated by PhenoAge may improve its sensitivity in identifying high‐risk populations. Furthermore, PhenoAge incorporates a broader range of clinical biomarkers, enabling a more comprehensive assessment of the health impacts resulting from physiological changes across different systems (Liu et al. 2018). In public health practice, merely determining whether aging is accelerated (i.e., a positive difference from chronological age) may be insufficient for risk assessment, as no current biological age metric can fully capture the progression of aging (Johnson and Shokhirev 2024). Therefore, we recommend also considering the extent of accelerated aging. Our dose–response relationship analysis further underscored this point, revealing that even modest increases in biological age can correlate with significantly heightened risks of multimorbidity and reduced LE. Different biological age assessment methods may emphasize varying aspects of the body's condition, suggesting that a combined approach utilizing multiple biological age metrics could be beneficial in practice (Belsky et al. 2018; Ferrucci et al. 2020).

Aging cannot be attributed solely to discrete molecular or cellular processes; rather, it results from disruptions in the intricate interactions among numerous biological processes occurring both within and across tissues (Cohen et al. 2022). Consequently, biological age should not be viewed as merely a reflection of a single biomarker, but rather as a complex outcome derived from the interplay of various physiological changes, chronic low‐grade inflammation, oxidative stress, and other multifaceted factors (Langenberg et al. 2023; Meier et al. 2023). These mechanisms collectively position biological age as a crucial indicator for assessing individual health risks and the progression of chronic diseases. With aging, the body's chronic low‐grade inflammatory responses become increasingly pronounced (Meier et al. 2023). The levels of inflammatory markers such as C‐reactive protein may rise as biological aging accelerates. These inflammatory markers are not only directly linked to a range of physical diseases, such as cardiovascular disease, diabetes (as indicated by glucose and glycated hemoglobin levels), and dementia but also contribute to the onset of chronic diseases, disability, frailty, and premature mortality, creating a positive feedback loop among multiple diseases (Ferrucci and Fabbri 2018). As an individual's age increases, factors such as cholesterol and blood pressure levels are associated with increased oxidative stress, leading to mechanisms such as vascular inflammation and endothelial dysfunction that contribute to cellular damage and death (Vatner et al. 2020). Oxidative damage affects major organs, including the heart, lungs, and liver, and has significant implications for brain cell health, as reflected by forced expiratory volume and vascular indicators (Barrows et al. 2019). Therefore, these insights strongly support the notion that biological age reflects the intricate interplay of physiological factors, significantly influencing overall health outcomes and chronic disease trajectories.

Our findings revealed a striking gradient: individuals with accelerated biological aging and physical–psychological–cognitive comorbidity at baseline experienced greater life expectancy loss compared to those without conditions. This suggests that accelerated aging may interacts synergistically with pre‐existing multimorbidity. This finding aligns with the literatures on biological aging and health deterioration (Duan et al. 2022; Jylhävä et al. 2017). From the perspective of biological mechanisms of aging, aging is the result of complex interactions between genetic, environmental, behavioral, and psychological risk factors (Kroemer et al. 2025). In simpler terms, with aging, the combined effects of structural changes in chromosomes, homeostenosis, and stem cell exhaustion lead to an accumulation of cellular waste at the microscopic level. When cellular damage exceeds the capacity for cellular regeneration, the organism begins to age, this process triggers functional disturbances at the cellular level, which subsequently result in the decline and dysfunction of organs and physiological systems (López‐Otín et al. 2023). Specifically, the ‘inflammaging’ framework posits that chronic low‐grade inflammation creates a permissive environment for tissue damage accumulation (Franceschi et al. 2018). Our KDM‐BA and PhenoAge models, which incorporates C‐reactive protein, may capture this feedforward loop where each additional disorder elevates systemic inflammation, thereby accelerating biological aging metrics disproportionately. Secondly, comorbidity imposes simultaneous energy demands on multiple organ systems (Miwa et al. 2022). Accelerated aging may reflect mitochondrial allostatic overload in different organs—a state where chronic oxidative stress exceeds cellular repair capacity (Miwa et al. 2022). This is particularly relevant given that both KDM‐BA and PhenoAge incorporates albumin and creatinine, biomarkers sensitive to mitochondrial efficiency. Finally, disease‐specific epigenetic reprogramming may create a permissive landscape for accelerated aging (Horvath and Raj 2018). The PhenoAge algorithm, originally trained on methylation sites predictive of mortality (Levine et al. 2018), captures this process through its incorporation of inflammatory and metabolic biomarkers. Importantly, in the sensitivity analysis, we found that individuals with shortened telomeres had a higher risk of multimorbidity and mortality (Figure S8, Table S11). This could stem from accelerated telomere attrition in progenitor cells, which may attempt to compensate for multiorgan functional decline.

As global public health has advanced, the survival rates of patients with common acute and chronic diseases have significantly improved, with the burden of disease increasingly shifting toward noncommunicable diseases (NCDs) (Li et al. 2020). Health behavior‐related diseases, such as cardiovascular diseases, cancers, chronic obstructive pulmonary disease, and diabetes, are becoming the predominant health burdens (Anderson and David Colby 2010; Tinetti et al. 2012). Nearly three‐quarters of individuals aged 65 and older are living with multiple chronic conditions, making them the primary consumers of healthcare services and accounting for more than two‐thirds of total healthcare spending (Vogeli et al. 2007). Those with multimorbidity tend to experience longer hospitalizations and a higher risk of premature mortality than individuals with single diseases do (Smith et al. 2021). This shift in the disease spectrum underscores the necessity for future public health policy development to better balance disease‐centered specialty care with patient‐centered primary care and community health systems, promoting a transition toward a “health‐centered” approach (The Academy of Medical Science 2018). Currently, primary care and community‐based multidisciplinary interventions are crucial approaches, yet higher‐quality evidence regarding their effectiveness in addressing multimorbidity remains essential (Smith et al. 2021). In this context, the concept of comprehensive geriatric assessment (CGA) based on multidisciplinary teams is valuable. CGA can comprehensively evaluate various dimensions of older individuals, including medical, psychological, cognitive, and environmental factors, demonstrating positive effects on improving outcomes and reducing hospitalization and mortality rates (Parker et al. 2017; Wu et al. 2024). Aging is a significant risk factor for multimorbidity; however, according to a large‐scale cross‐sectional survey, while the prevalence of multimorbidity is markedly higher in those aged 65 years and older, the sheer size of the younger population means that there are more individuals under 65 years living with multimorbidity than in the older cohort (Barnett et al. 2012). If public health intervention strategies are solely based on chronological age (i.e., focusing on older adults), this may overlook a larger, more cost‐effective group for intervention. The cumulative effects of health throughout life also impact the biological age of middle‐aged and older adults (Cui et al. 2024; Yu et al. 2024). Compared with chronological age, biological age more accurately reflects an individual's experiences throughout their life, and is influenced by genes, the environment, and health behaviors (Oblak et al. 2021). In fact, more than 50% of premature deaths attributable to noncommunicable diseases are preventable (Jamison et al. 2024; Martinez et al. 2020). The presence of physical illnesses is a significant risk factor for the development of cognitive and psychological disorders; whether or not chronic conditions exist, maintaining a healthy lifestyle can help extend life expectancy (Chudasama et al. 2020, 2019). In public health and clinical practice, the biological age serves as a valuable tool for identifying high‐risk populations, aiding in optimizing the allocation of health management resources. Particularly in resource‐limited settings, targeting interventions in groups of greater biological age can effectively increase the efficiency of health management efforts.

This study revealed a significant association between accelerated biological aging and multimorbidity, highlighting the potential value of biological age in identifying high‐risk populations. However, certain limitations must be acknowledged. First, the research is based on data from the UK Biobank, which, while representative, primarily draws from specific populations that may affect the generalizability of the results, particularly among individuals from diverse racial, socioeconomic, and geographical backgrounds. Second, while the study employed both retrospective and prospective analyses, the complex time sequences and interactions of different diseases necessitate further longitudinal research to establish causal relationships. Third, different types of chronic diseases, as well as their varying durations and severities, have distinct impacts on the occurrence of multimorbidity and health outcomes. Combining them within the same framework may understate the effects of certain severe diseases (Griffith et al. 2019). Finally, reliance on self‐reported health status and lifestyle data may introduce reporting bias, potentially affecting the accuracy of the findings. Additionally, while cognitive disorders were assessed at baseline using standardized scales, their evaluation during follow‐up relied solely on diagnostic records, which may have led to an underestimation of the incidence of cognitive disorders over time. These limitations underscore the need for future research to utilize broader and more diverse datasets, incorporating additional biomarkers and objective measurements to achieve a more comprehensive understanding of the relationship between biological aging and multimorbidity.

## 5 Conclusions

In conclusion, this study provides an in‐depth examination of the association between accelerated biological aging and multimorbidity, including physical, psychological, and cognitive disorders, revealing the significant potential of biological age as a tool for assessing health risks. This finding underscores the critical role of biological aging as a predictor of multimorbidity. By utilizing this metric, public health strategies can implement more effective interventions to address the complex challenges posed by multiple chronic diseases. Ultimately, this approach has the potential to improve overall health outcomes by effectively identifying individual health risks, thereby contributing to a healthier aging population and fostering more sustainable healthcare systems by alleviating the burden of chronic diseases and associated healthcare costs.

## Author Contributions

Z.T. conceptualized the study, conducted data analysis, and drafted the manuscript. Z.R. and B.S. contributed to study conceptualization and methodological development, with Z.R. revising the initial draft. C.C. and Y.L. facilitated variable extraction, index construction, and ensured reproducibility while contributing to manuscript revisions. S.G. provided methodological guidance and participated in manuscript refinement. Y.W. collaborated on manuscript conceptualization and revisions. X.Z. oversaw study design, secured funding, provided supervision, and led manuscript revisions. All authors read and approved the final manuscript.

## Ethics Statement

The UK Biobank study received ethical approval from the North West Multi‐centre Research Ethics Committee (REC reference: 21/NW/0157). All participants provided written informed consent to participate in UK Biobank.

## Conflicts of Interest

The authors declare no conflicts of interest.

## Supporting information


Data S1.

## Data Availability

The data supporting this study's findings are available through the UK Biobank resource (https://www.ukbiobank.ac.uk) under Application Number 105435. Access requires registration and project approval via the standard UK Biobank access procedure.

## References

[acel70142-bib-0001] Anderson, G. , and P. D. David Colby . 2010. “Chronic Care: Making the Case for Ongoing Care.”

[acel70142-bib-0002] Barnett, K. , S. W. Mercer , M. Norbury , G. Watt , S. Wyke , and B. Guthrie . 2012. “Epidemiology of Multimorbidity and Implications for Health Care, Research, and Medical Education: A Cross‐Sectional Study.” Lancet 380, no. 9836: 37–43. 10.1016/s0140-6736(12)60240-2. 22579043

[acel70142-bib-0003] Barrows, I. R. , A. Ramezani , and D. S. Raj . 2019. “Inflammation, Immunity, and Oxidative Stress in Hypertension‐Partners in Crime?” Advances in Chronic Kidney Disease 26, no. 2: 122–130. 10.1053/j.ackd.2019.03.001. 31023446 PMC6816256

[acel70142-bib-0004] Belsky, D. W. , T. E. Moffitt , A. A. Cohen , et al. 2018. “Eleven Telomere, Epigenetic Clock, and Biomarker‐Composite Quantifications of Biological Aging: Do They Measure the Same Thing?” American Journal of Epidemiology 187, no. 6: 1220–1230. 10.1093/aje/kwx346. 29149257 PMC6248475

[acel70142-bib-0005] Chen, L. , B. Wu , L. Mo , et al. 2024. “Associations Between Biological Ageing and the Risk of, Genetic Susceptibility to, and Life Expectancy Associated With Rheumatoid Arthritis: A Secondary Analysis of Two Observational Studies.” Lancet Healthy Longevity 5, no. 1: e45–e55. 10.1016/s2666-7568(23)00220-9. 38081205

[acel70142-bib-0006] Chudasama, Y. V. , K. Khunti , C. L. Gillies , et al. 2020. “Healthy Lifestyle and Life Expectancy in People With Multimorbidity in the UK Biobank: A Longitudinal Cohort Study.” PLoS Medicine 17, no. 9: e1003332. 10.1371/journal.pmed.1003332. 32960883 PMC7508366

[acel70142-bib-0007] Chudasama, Y. V. , K. K. Khunti , F. Zaccardi , et al. 2019. “Physical Activity, Multimorbidity, and Life Expectancy: A UK Biobank Longitudinal Study.” BMC Medicine 17, no. 1: 108. 10.1186/s12916-019-1339-0. 31186007 PMC6560907

[acel70142-bib-0008] Cohen, A. A. , L. Ferrucci , T. Fülöp , et al. 2022. “A Complex Systems Approach to Aging Biology.” Nature Aging 2, no. 7: 580–591. 10.1038/s43587-022-00252-6. 37117782 PMC12007111

[acel70142-bib-0009] Cui, F. , L. Tang , D. Li , et al. 2024. “Early‐Life Exposure to Tobacco, Genetic Susceptibility, and Accelerated Biological Aging in Adulthood.” Science Advances 10, no. 18: eadl3747. 10.1126/sciadv.adl3747. 38701212 PMC11068008

[acel70142-bib-0010] Duan, R. , Q. Fu , Y. Sun , and Q. Li . 2022. “Epigenetic Clock: A Promising Biomarker and Practical Tool in Aging.” Ageing Research Reviews 81: 101743. 10.1016/j.arr.2022.101743. 36206857

[acel70142-bib-0011] DuGoff, E. H. , V. Canudas‐Romo , C. Buttorff , B. Leff , and G. F. Anderson . 2014. “Multiple Chronic Conditions and Life Expectancy: A Life Table Analysis.” Medical Care 52, no. 8: 688–694. 10.1097/mlr.0000000000000166. 25023914

[acel70142-bib-0012] Ferrucci, L. , and E. Fabbri . 2018. “Inflammageing: Chronic Inflammation in Ageing, Cardiovascular Disease, and Frailty.” Nature Reviews. Cardiology 15, no. 9: 505–522. 10.1038/s41569-018-0064-2. 30065258 PMC6146930

[acel70142-bib-0013] Ferrucci, L. , M. Gonzalez‐Freire , E. Fabbri , et al. 2020. “Measuring Biological Aging in Humans: A Quest.” Aging Cell 19, no. 2: e13080. 10.1111/acel.13080. 31833194 PMC6996955

[acel70142-bib-0014] Franceschi, C. , P. Garagnani , P. Parini , C. Giuliani , and A. Santoro . 2018. “Inflammaging: A New Immune‐Metabolic Viewpoint for Age‐Related Diseases.” Nature Reviews. Endocrinology 14, no. 10: 576–590. 10.1038/s41574-018-0059-4. 30046148

[acel70142-bib-0015] Gao, X. , T. Geng , M. Jiang , et al. 2023. “Accelerated Biological Aging and Risk of Depression and Anxiety: Evidence From 424,299 UK Biobank Participants.” Nature Communications 14, no. 1: 2277. 10.1038/s41467-023-38013-7. PMC10119095 37080981

[acel70142-bib-0016] GBD 2021 Causes of Death Collaborators . 2024. “Global Burden of 288 Causes of Death and Life Expectancy Decomposition in 204 Countries and Territories and 811 Subnational Locations, 1990–2021: A Systematic Analysis for the Global Burden of Disease Study 2021.” Lancet 403, no. 10440: 2100–2132. 10.1016/s0140-6736(24)00367-2. 38582094 PMC11126520

[acel70142-bib-0017] Griffith, L. E. , A. Gilsing , D. Mangin , et al. 2019. “Multimorbidity Frameworks Impact Prevalence and Relationships With Patient‐Important Outcomes.” Journal of the American Geriatrics Society 67, no. 8: 1632–1640. 10.1111/jgs.15921. 30957230

[acel70142-bib-0018] Horvath, S. , and K. Raj . 2018. “DNA Methylation‐Based Biomarkers and the Epigenetic Clock Theory of Ageing.” Nature Reviews. Genetics 19, no. 6: 371–384. 10.1038/s41576-018-0004-3. 29643443

[acel70142-bib-0019] Jackson, C. 2011. “Multi‐State Models for Panel Data: The Msm Package for R.” Journal of Statistical Software 38, no. 8: 1–28. 10.18637/jss.v038.i08.

[acel70142-bib-0020] Jamison, D. T. , L. H. Summers , A. Y. Chang , et al. 2024. “Global Health 2050: The Path to Halving Premature Death by Mid‐Century.” Lancet 404, no. 10462: 1561–1614. 10.1016/s0140-6736(24)01439-9. 39419055 PMC12320876

[acel70142-bib-0021] Jiang, M. , S. Tian , S. Liu , et al. 2024. “Accelerated Biological Aging Elevates the Risk of Cardiometabolic Multimorbidity and Mortality.” Nature Cardiovascular Research 3, no. 3: 332–342. 10.1038/s44161-024-00438-8. PMC13265155 39196113

[acel70142-bib-0022] Johnson, A. A. , and M. N. Shokhirev . 2024. “Contextualizing Aging Clocks and Properly Describing Biological Age.” Aging Cell 23, no. 12: e14377. 10.1111/acel.14377. 39392224 PMC11634725

[acel70142-bib-0023] Jylhävä, J. , N. L. Pedersen , and S. Hägg . 2017. “Biological Age Predictors.” eBioMedicine 21: 29–36. 10.1016/j.ebiom.2017.03.046. 28396265 PMC5514388

[acel70142-bib-0024] Klemera, P. , and S. Doubal . 2006. “A New Approach to the Concept and Computation of Biological Age.” Mechanisms of Ageing and Development 127, no. 3: 240–248. 10.1016/j.mad.2005.10.004. 16318865

[acel70142-bib-0025] Kroemer, G. , A. B. Maier , A. M. Cuervo , et al. 2025. “From Geroscience to Precision Geromedicine: Understanding and Managing Aging.” Cell 188, no. 8: 2043–2062. 10.1016/j.cell.2025.03.011. 40250404 PMC12037106

[acel70142-bib-0026] Kwon, D. , and D. W. Belsky . 2021. “A Toolkit for Quantification of Biological Age From Blood Chemistry and Organ Function Test Data: BioAge.” Geroscience 43, no. 6: 2795–2808. 10.1007/s11357-021-00480-5. 34725754 PMC8602613

[acel70142-bib-0027] Lambert, P. C. , and P. Royston . 2009. “Further Development of Flexible Parametric Models for Survival Analysis.” Stata Journal 9: 265–290.

[acel70142-bib-0028] Langenberg, C. , A. D. Hingorani , and C. J. M. Whitty . 2023. “Biological and Functional Multimorbidity‐From Mechanisms to Management.” Nature Medicine 29, no. 7: 1649–1657. 10.1038/s41591-023-02420-6. 37464031

[acel70142-bib-0029] Levine, M. E. , A. T. Lu , A. Quach , et al. 2018. “An Epigenetic Biomarker of Aging for Lifespan and Healthspan.” Aging (Albany NY) 10, no. 4: 573–591. 10.18632/aging.101414. 29676998 PMC5940111

[acel70142-bib-0030] Li, Y. , J. Schoufour , D. D. Wang , et al. 2020. “Healthy Lifestyle and Life Expectancy Free of Cancer, Cardiovascular Disease, and Type 2 Diabetes: Prospective Cohort Study.” BMJ (Clinical Research Ed) 368: l6669. 10.1136/bmj.l6669. PMC7190036 31915124

[acel70142-bib-0031] Liu, Z. , P. L. Kuo , S. Horvath , E. Crimmins , L. Ferrucci , and M. Levine . 2018. “A New Aging Measure Captures Morbidity and Mortality Risk Across Diverse Subpopulations From NHANES IV: A Cohort Study.” PLoS Medicine 15, no. 12: e1002718. 10.1371/journal.pmed.1002718. 30596641 PMC6312200

[acel70142-bib-0032] López‐Otín, C. , M. A. Blasco , L. Partridge , M. Serrano , and G. Kroemer . 2023. “Hallmarks of Aging: An Expanding Universe.” Cell 186, no. 2: 243–278. 10.1016/j.cell.2022.11.001. 36599349

[acel70142-bib-0033] Mak, J. K. L. , C. E. McMurran , R. Kuja‐Halkola , et al. 2023. “Clinical Biomarker‐Based Biological Aging and Risk of Cancer in the UK Biobank.” British Journal of Cancer 129, no. 1: 94–103. 10.1038/s41416-023-02288-w. 37120669 PMC10307789

[acel70142-bib-0034] Martinez, R. , P. Lloyd‐Sherlock , P. Soliz , et al. 2020. “Trends in Premature Avertable Mortality From Non‐Communicable Diseases for 195 Countries and Territories, 1990–2017: A Population‐Based Study.” Lancet Global Health 8, no. 4: e511–e523. 10.1016/s2214-109x(20)30035-8. 32199120

[acel70142-bib-0035] Meier, H. C. S. , C. Mitchell , T. Karadimas , and J. D. Faul . 2023. “Systemic Inflammation and Biological Aging in the Health and Retirement Study.” Geroscience 45, no. 6: 3257–3265. 10.1007/s11357-023-00880-9. 37501048 PMC10643484

[acel70142-bib-0036] Menotti, A. , I. Mulder , A. Nissinen , S. Giampaoli , E. J. Feskens , and D. Kromhout . 2001. “Prevalence of Morbidity and Multimorbidity in Elderly Male Populations and Their Impact on 10‐Year All‐Cause Mortality: The FINE Study (Finland, Italy, Netherlands, Elderly).” Journal of Clinical Epidemiology 54, no. 7: 680–686. 10.1016/s0895-4356(00)00368-1. 11438408

[acel70142-bib-0037] Miwa, S. , S. Kashyap , E. Chini , and T. von Zglinicki . 2022. “Mitochondrial Dysfunction in Cell Senescence and Aging.” Journal of Clinical Investigation 132, no. 13: e158447. 10.1172/jci158447. 35775483 PMC9246372

[acel70142-bib-0038] Ni, Y. , Y. Zhou , M. Kivimäki , et al. 2023. “Socioeconomic Inequalities in Physical, Psychological, and Cognitive Multimorbidity in Middle‐Aged and Older Adults in 33 Countries: A Cross‐Sectional Study.” Lancet Healthy Longevity 4, no. 11: e618–e628. 10.1016/s2666-7568(23)00195-2. 37924843

[acel70142-bib-0039] Oblak, L. , J. van der Zaag , A. T. Higgins‐Chen , M. E. Levine , and M. P. Boks . 2021. “A Systematic Review of Biological, Social and Environmental Factors Associated With Epigenetic Clock Acceleration.” Ageing Research Reviews 69: 101348. 10.1016/j.arr.2021.101348. 33930583

[acel70142-bib-0040] Parker, S. G. , A. McLeod , P. McCue , et al. 2017. “New Horizons in Comprehensive Geriatric Assessment.” Age and Ageing 46, no. 5: 713–721. 10.1093/ageing/afx104. 28874007

[acel70142-bib-0041] Piening, B. D. , J. Lovejoy , and J. C. Earls . 2020. “Ageotypes: Distinct Biomolecular Trajectories in Human Aging.” Trends in Pharmacological Sciences 41, no. 5: 299–301. 10.1016/j.tips.2020.02.003. 32192755

[acel70142-bib-0042] Skou, S. T. , F. S. Mair , M. Fortin , et al. 2022. “Multimorbidity.” Nature Reviews Disease Primers 8, no. 1: 48. 10.1038/s41572-022-00376-4. PMC7613517 35835758

[acel70142-bib-0043] Smith, S. M. , E. Wallace , T. O'Dowd , and M. Fortin . 2021. “Interventions for Improving Outcomes in Patients With Multimorbidity in Primary Care and Community Settings.” Cochrane Database of Systematic Reviews 1, no. 1: Cd006560. 10.1002/14651858.CD006560.pub4. 33448337 PMC8092473

[acel70142-bib-0044] Sudlow, C. , J. Gallacher , N. Allen , et al. 2015. “UK Biobank: An Open Access Resource for Identifying the Causes of a Wide Range of Complex Diseases of Middle and Old Age.” PLoS Medicine 12, no. 3: e1001779. 10.1371/journal.pmed.1001779. 25826379 PMC4380465

[acel70142-bib-0045] Talifu, Z. , S. Guo , B. Su , et al. 2024. “Gender Disparities in Multi‐State Health Transitions and Life Expectancy Among the ≥50‐Year‐Old Population: A Cross‐National Multi‐Cohort Study.” Journal of Global Health 14: 04156. 10.7189/jogh.14.04156. 39238364 PMC11377966

[acel70142-bib-0046] The Academy of Medical Science . 2018. “Multimorbidity: A Priority for Global Health Research.”

[acel70142-bib-0047] Tinetti, M. E. , T. R. Fried , and C. M. Boyd . 2012. “Designing Health Care for the Most Common Chronic Condition—Multimorbidity.” JAMA 307, no. 23: 2493–2494. 10.1001/jama.2012.5265. 22797447 PMC4083627

[acel70142-bib-0048] Vatner, S. F. , J. Zhang , M. Oydanich , T. Berkman , R. Naftalovich , and D. E. Vatner . 2020. “Healthful Aging Mediated by Inhibition of Oxidative Stress.” Ageing Research Reviews 64: 101194. 10.1016/j.arr.2020.101194. 33091597 PMC7710569

[acel70142-bib-0049] Vogeli, C. , A. E. Shields , T. A. Lee , et al. 2007. “Multiple Chronic Conditions: Prevalence, Health Consequences, and Implications for Quality, Care Management, and Costs.” Journal of General Internal Medicine 22, no. Suppl 3: 391–395. 10.1007/s11606-007-0322-1. 18026807 PMC2150598

[acel70142-bib-0050] Wu, Z. , J. Qu , and G. H. Liu . 2024. “Roles of Chromatin and Genome Instability in Cellular Senescence and Their Relevance to Ageing and Related Diseases.” Nature Reviews. Molecular Cell Biology 25, no. 12: 979–1000. 10.1038/s41580-024-00775-3. 39363000

[acel70142-bib-0051] Yu, J. , F. Pu , G. Yang , et al. 2024. “Sex‐Specific Association Between Childhood Adversity and Accelerated Biological Aging.” Advanced Science 11, no. 26: e2309346. 10.1002/advs.202309346. 38704685 PMC11234451

[acel70142-bib-0052] Yusri, K. , S. Kumar , S. Fong , J. Gruber , and V. Sorrentino . 2024. “Towards Healthy Longevity: Comprehensive Insights From Molecular Targets and Biomarkers to Biological Clocks.” International Journal of Molecular Sciences 25, no. 12: 6793. 10.3390/ijms25126793. 38928497 PMC11203944

[acel70142-bib-0053] Zheng, G. , Q. Chang , Y. Zhang , et al. 2024. “Associations of Clinical Parameter‐Based Accelerated Aging, Genetic Predisposition With Risk of Chronic Kidney Disease and Associated Life Expectancy: A Prospective Cohort Study.” Aging Cell 24: e14453. 10.1111/acel.14453. 39717880 PMC11984662

[acel70142-bib-0054] Zhou, Y. , X. Dai , Y. Ni , et al. 2023. “Interventions and Management on Multimorbidity: An Overview of Systematic Reviews.” Ageing Research Reviews 87: 101901. 10.1016/j.arr.2023.101901. 36905961

[acel70142-bib-0055] Zhou, Y. , M. Kivimäki , L. L. Yan , et al. 2024. “Associations Between Socioeconomic Inequalities and Progression to Psychological and Cognitive Multimorbidities After Onset of a Physical Condition: A Multicohort Study.” EClinicalMedicine 74: 102739. 10.1016/j.eclinm.2024.102739. 39157288 PMC11327438

