# Supplementary material for: The Association Between Accelerated Biological Aging and the Physical, Psychological, and Cognitive Multimorbidity and Life Expectancy: Cohort Study
Source: Aging Cell. 2025 Jul 13;24(9):e70142. doi: 10.1111/acel.70142 (PMC12419857; doi:10.1111/acel.70142)
Supplement: Supplementary file 1 — Data S1. [file ACEL-24-e70142-s001.docx]

**The association between accelerated biological aging and the physical, psychological, and cognitive multimorbidity and life expectancy: cohort study**

**Supplementary**

**Supplementary Figures:**

Figure S1. Flowchart of participant selection and analysis

Figure S2. Comparison of data distribution intensity before and after imputation of key covariates

Figure S3. Schematic diagram of Markov model for state transition of single disorder, comorbidity and death

Figure S4. Comorbidity patterns and mortality at baseline and during follow-up

Figure S5. Association between Accelerated Biological Aging and Mortality Risk During Follow-Up Under Different Baseline Conditions

Figure S6. Dose-response relationships of KDM-BA and PhenoAge accelerations with risks of mortality during follow-up

Figure S7. Impact of accelerated biological aging on life expectancy by health status

Figure S8. Dose-response relationships of KDM-BA and PhenoAge accelerations with risks of single disorder, comorbidity and mortality during follow-up in baseline disorder-free population (excluding baseline and early-onset disorders)

Figure S9. Dose-response relationships of telomere length with risks of multimorbidity outcomes and mortality during follow-up

Figure S10. Impact of shortened telomere length on life expectancy by health status

**Supplementary Tables:**

Table S1. Categorization of physical, psychological, and cognitive disorders with ICD-10 codes and testing.

Table S2. Cognitive tests included in each cognitive domain.

Table S3. Variables for the construction of biological ages

Table S4. Variables used to calculate healthy diet score in the UK biobank

Table S5. Association between accelerated aging and multimorbidity involving physical, psychological, and cognitive disorders at baseline

Table S6. Association between accelerated aging at base line with incident multimorbidity involving physical, psychological, and cognitive disorders at follow-up

Table S7. Characteristics of study participants according to health status

Table S8. Association between accelerated aging and multimorbidity involving physical, psychological, and cognitive disorders at baseline with complete covariates

Table S9. Association between accelerated aging and multimorbidity involving physical, psychological, and cognitive disorders at follow-up with complete covariates

Table S10. of accelerated aging with follow-up multimorbidity excluding baseline and early-onset disorders

Table S11. Association between telomere length and multimorbidity at baseline

Table S12. Association between telomere length and multimorbidity during follow-up

**Figure S1. Flowchart of participant selection and analysis**

Note. * Extreme values were defined as KDM-BA/PhenoAge measurements beyond ±5 standard deviations (SD) from the cohort mean.


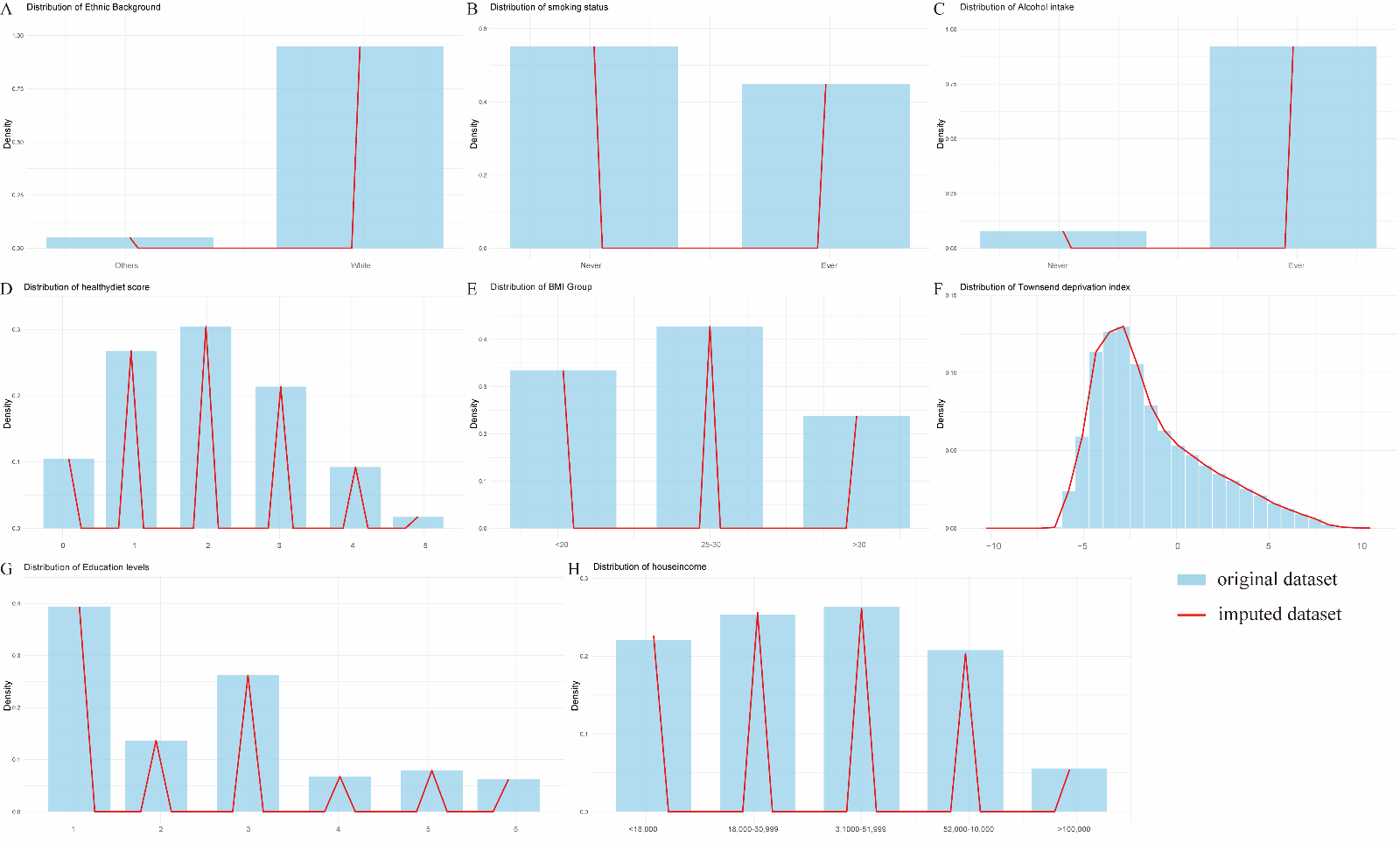


**Figure S2. Comparison of data distribution intensity before and after imputation of key covariates.**


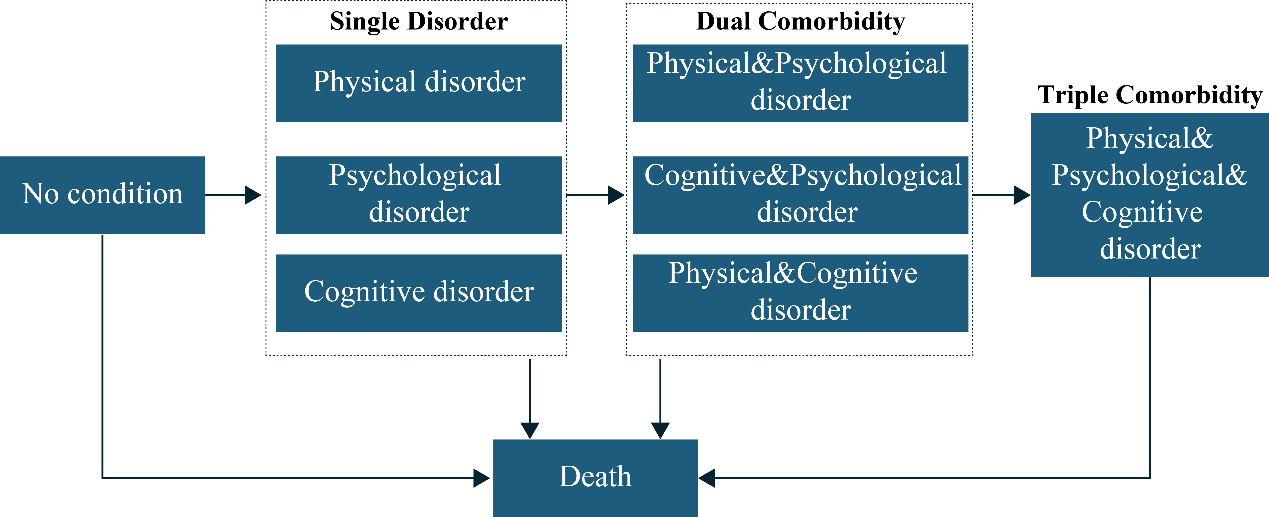


**Figure S3. Schematic diagram of Markov model for state transition of single disorder, comorbidity and death**


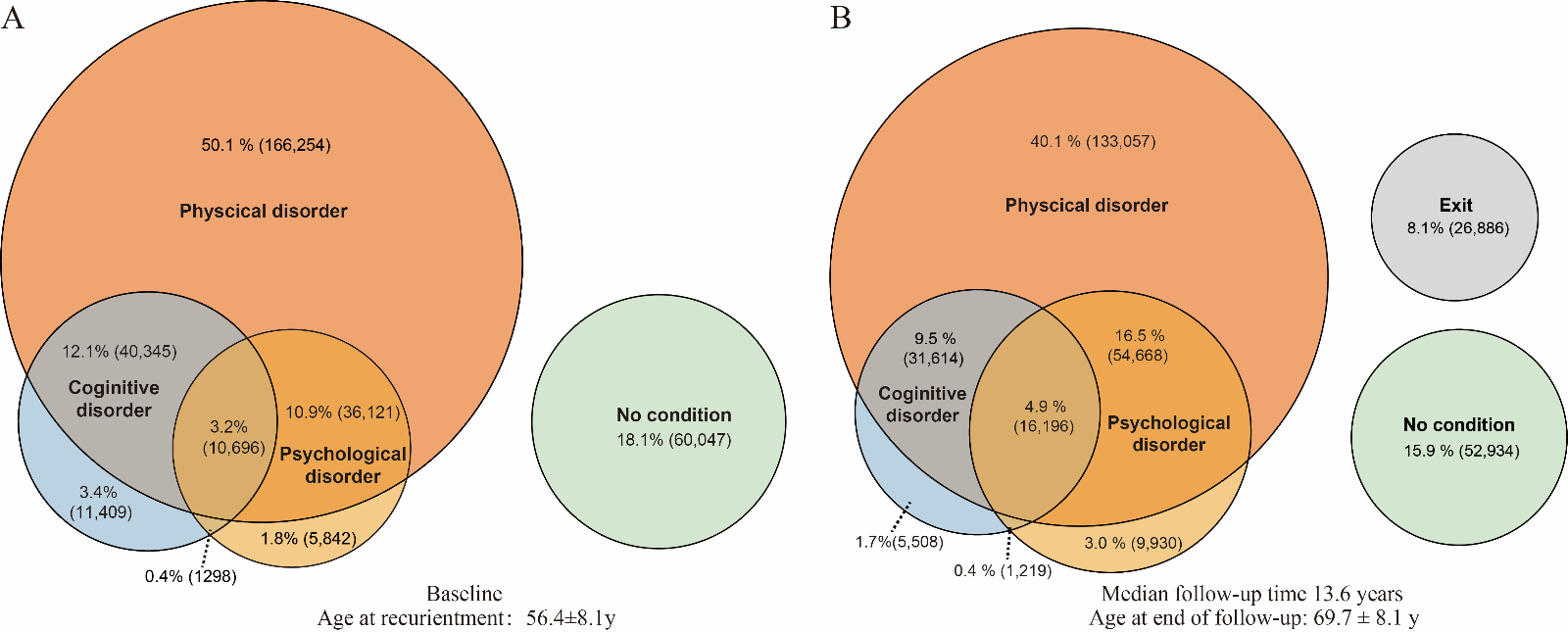


**Figure S4. Comorbidity patterns and mortality at baseline and during follow-up**

**
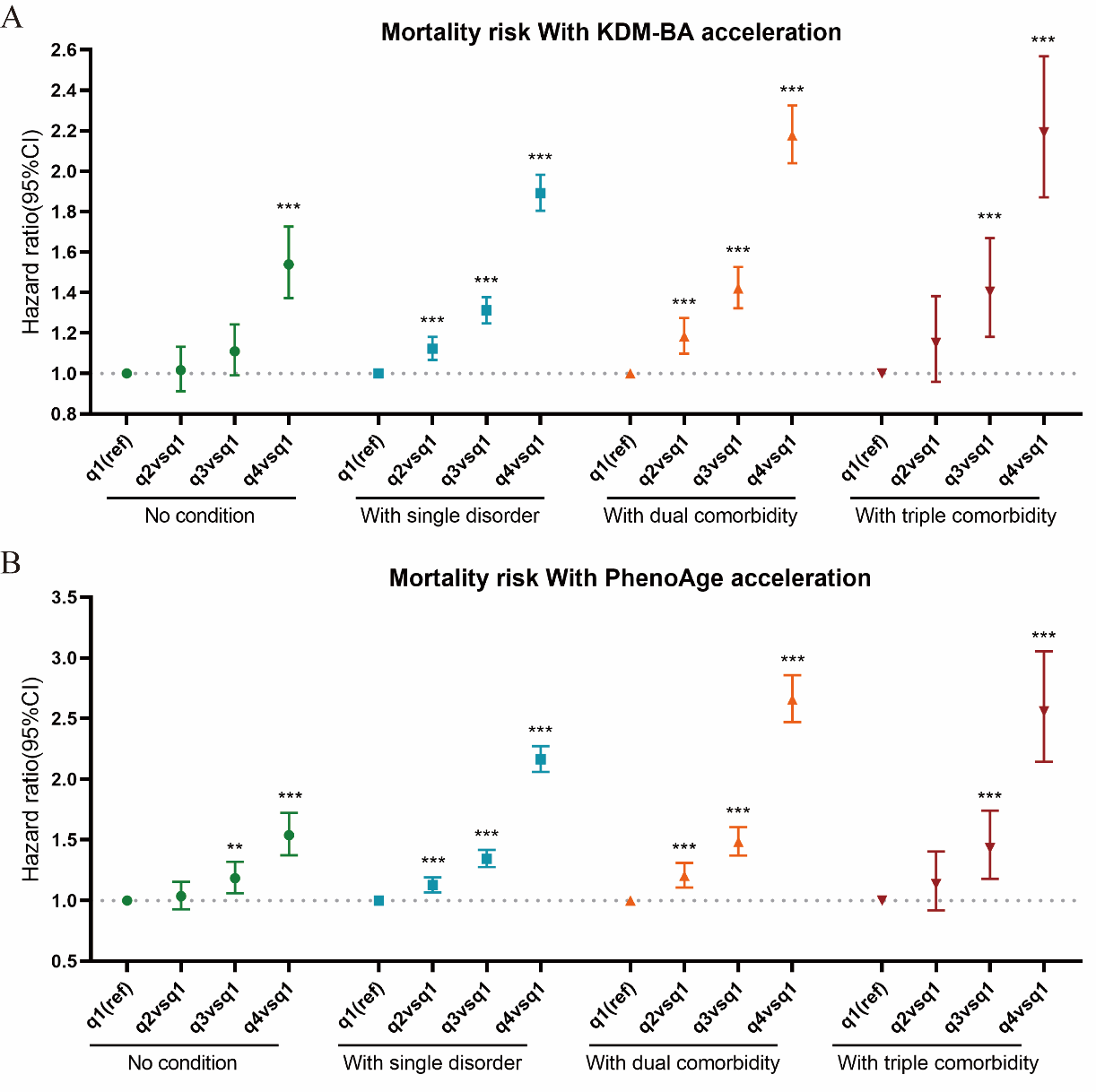
**

**Figure S5. Association between Accelerated Biological Aging and Mortality Risk During Follow-Up Under Different Baseline Conditions**

Note: Models were adjusted for age, sex, ethnicity, BMI, smoking status, alcohol intake, healthy diet score, physical activity, Townsend deprivation index, household income, and air pollution.

**
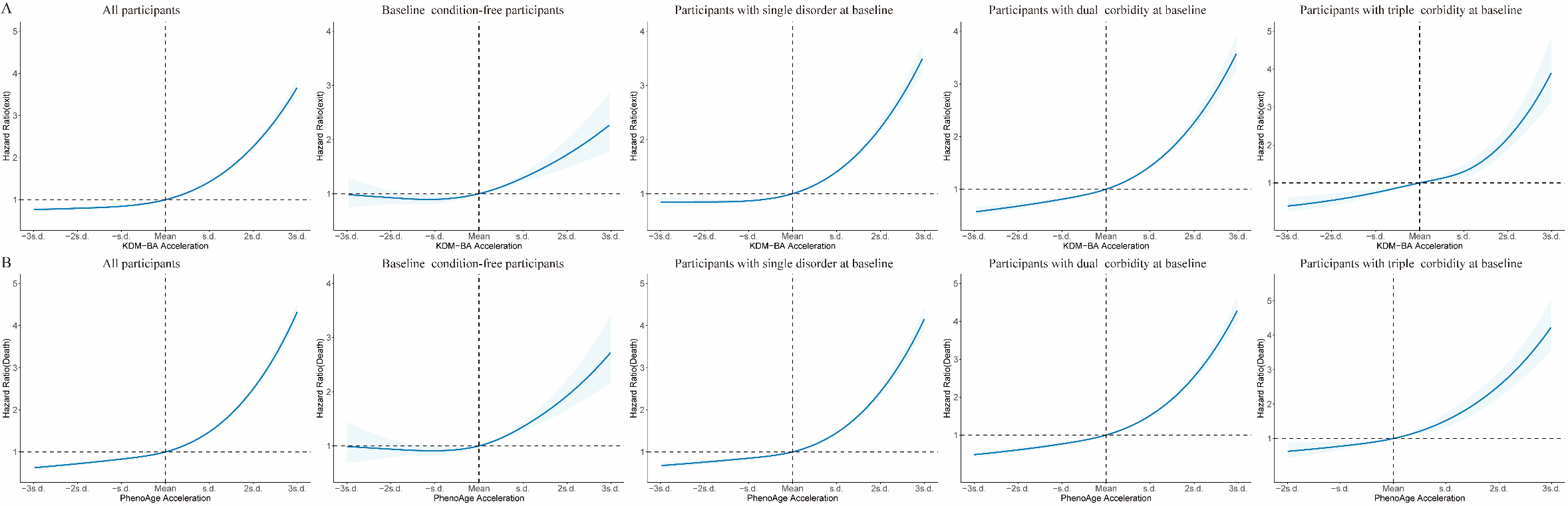
**

**Figure S6. Dose-response relationships of KDM-BA and PhenoAge accelerations with risks of mortality during follow-up.** (A) Association of KDM-BA acceleration with the risk of mortality across different baseline health status groups. (B) Association of PhenoAge acceleration with the risk of mortality across different baseline health status groups..

Models were adjusted for age, sex, ethnicity, BMI, smoking status, alcohol intake, healthy diet score, physical activity, Townsend deprivation index, household income, and air pollution.

***
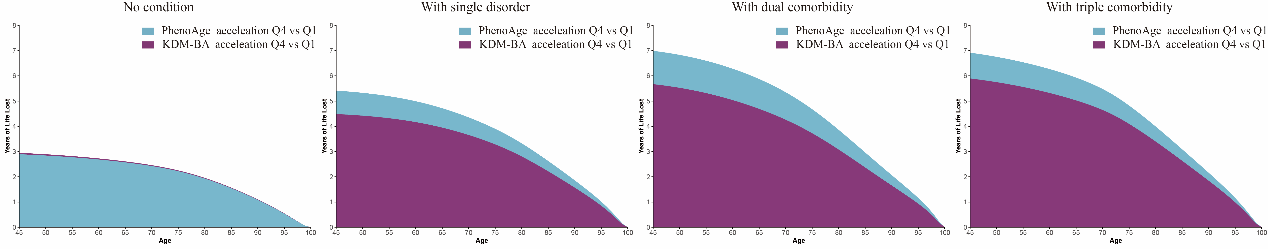
***

**Figure S7. Impact of accelerated biological aging on life expectancy by health status**

The models were adjusted for age, sex, ethnicity, BMI, smoking status, alcohol intake, healthy diet score, physical activity, Townsend deprivation index, household income, and air pollution.


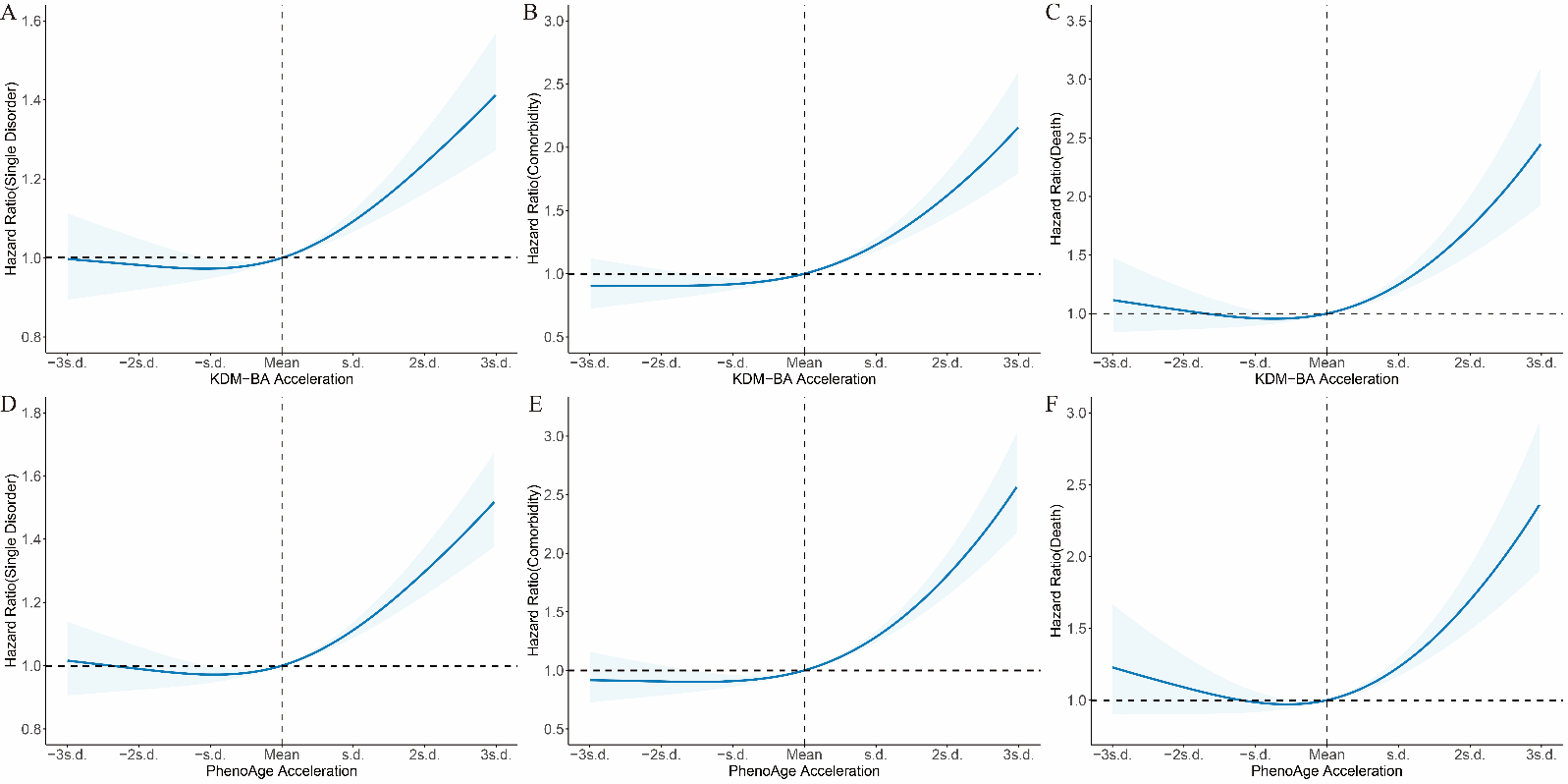


**Figure S8. Dose-response relationships of KDM-BA and PhenoAge accelerations with risks of single disorder, comorbidity and mortailty during follow-up in baseline disorder-free population (excluding baseline and early-onset disorders*).**

Note. * Excluding individuals with disorders at baseline and those who developed disorders or died within the first 5 years of follow-up.

The models have been adjusted for age, sex, ethnicity, BMI, smoking status, alcohol intake, healthy diet score, physical activity, Townsend deprivation index, household income, and air pollution.


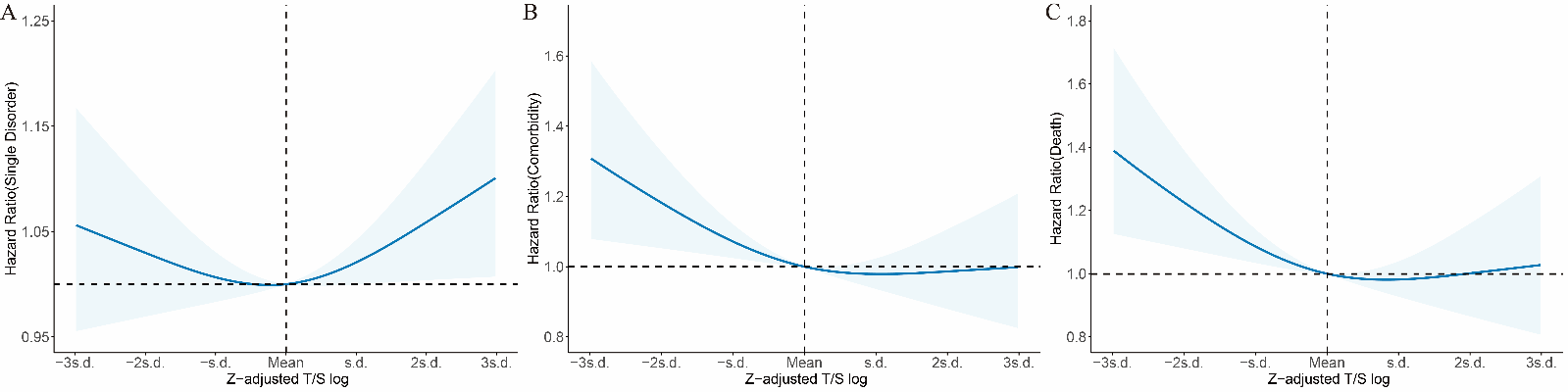


**Figure S9. Dose-response relationships of telomere length with risks of multimorbidity outcomes and mortality during follow-up.** (A) Single disorder, (B) Dual and Triple Comorbidity, (C)Death.

Models were adjusted for age, sex, ethnicity, BMI, smoking status, alcohol intake, healthy diet score, physical activity, Townsend deprivation index, household income, and air pollution.


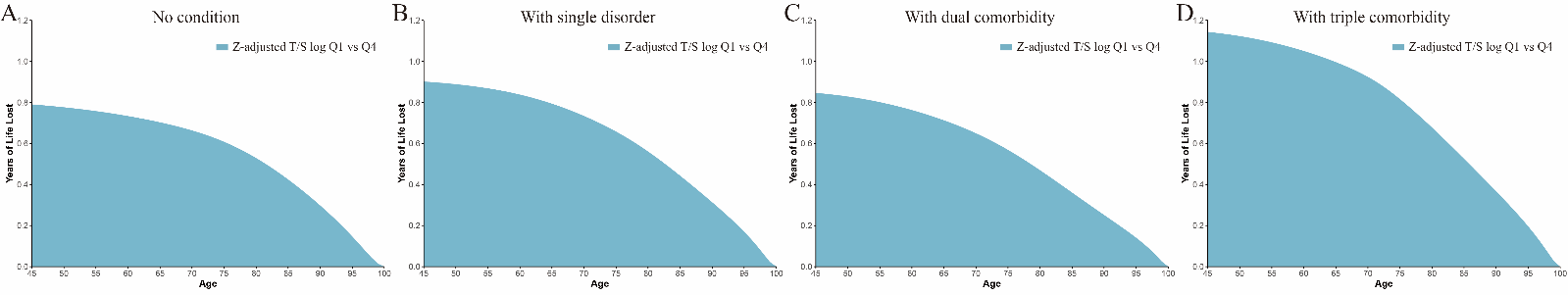


**Figure S10. Impact of shortened telomere length on life expectancy by health status.** (A) Single disorder, (B) Dual Comorbidity, (C) Triple Comorbidity and (D)Death.

Models were adjusted for age, sex, ethnicity, BMI, smoking status, alcohol intake, healthy diet score, physical activity, Townsend deprivation index, household income, and air pollution.

**Table S1. Categorization of physical, psychological, and cognitive disorders with ICD-10 codes and testing.**

|  | **Condition** | **UK Biobank coding (data field 20002)** | **ICD10 code** |
| --- | --- | --- | --- |
| Physical disorder^2^ | Asthma | 1111 Asthma | J45 Asthma |
|  | Atrial fibrillation | 1471 Atrial fibrillation | I48 Atrial fibrillation and flutter  I49 Other cardiac arrythmias |
|  | Bronchiectasis | 1114 Bronchiectasis | J47 Bronchiectasis |
|  | Cancer | UK Biobank data field 2453 | C00-C97 Malignant neoplasms |
|  | Chronic fatigue syndrome (CFS) | 1482 Chronic fatigue syndrome | R53 Malaise and fatigue |
|  | Chronic kidney disease (CKD) | 1192 Renal/kidney failure  1193 Renal failure requiring dialysis  1194 Renal failure not requiring dialysis  1427 Polycystic kidney  1519 Kidney nephropathy  1520 IGA nephropathy  1607 Diabetic nephropathy | N00 Acute nephritic syndrome  N01 Rapidly progressive nephritic syndrome  N03 Chronic nephritic syndrome  N04 Nephrotic syndrome  N05 Unspecified nephritic syndrome  N08 Glomerular disorders in diseases classified elsewhere  N17 Acute kidney failure  N18 Chronic renal failure  N19 Unspecified kidney failure  E10.2 Type 1 diabetes mellitus with diabetic chronic kidney disease  E11.2 Type 2 diabetes mellitus with diabetic chronic kidney disease  E13.2 Other specified diabetes mellitus with diabetic chronic kidney disease  I12 Hypertensive renal disease  I13 Hypertensive heart and renal disease |
|  | Chronic obstructive pulmonary disorder (COPD) | 1112 Chronic obstructive airways disease/COPD  1113 Emphysema/chronic bronchitis  1472 Emphysema | J40 Bronchitis, not specified as acute or chronic  J41 Simple and mucopurulent chronic bronchitis  J42 Unspecified chronic bronchitis  J43 Emphysema  J44 Other chronic obstructive pulmonary disease |
|  | Chronic sinusitis | 1416 Chronic sinusitis | J32 Chronic sinustitis |
|  | Connective tissue disorders | 1322 Myositis/myopathy  1373 Connective tissue disorder  1377 Polymyalgia rheumatica  1381 Systemic lupus erythematosis/ SLE  1382 Sjogren’s syndrome/sicca syndrome  1383 Dermatopolymyositis  1384 Scleroderma/systemic sclerosis  1456 Malabsorption/coeliac disease  1464 Rheumatoid arthritis  1477 Psoriatic arthropathy  1480 Dermatomyositis  1481 Polymyositis | M05 Rheumatoid arthritis  M06 Other rheumatoid arthritis  M07 Psoriatic and enteropathic arthropathies  M08 Juvenile arthritis  M30 Polyarteritis nodosa and related conditions  M31 Other necrotizing vasculopathies  M32 Systemic lupus erythematosus  M33 Dermatopolymyositis  M34 Systemic sclerosis  M35 Other systemic involvement of connective tissue  M36 Systemic disorders of connective tissue in diseases classified elsewhere  M60 Myositis  K90 Intestinal malabsorption/coeliac disease |
|  | Coronary heart disease (CHD) | 1074 Angina  1075 Heart attack/myocardial infarction | I20 Angina pectoris  I21 Acute myocardial infarction  I22 Subsequent myocardial infarction  I23 Certain current complications following acute myocardial infarction  I24 Other acute ischaemic heart diseases  I25 Chronic ischaemic heart disease |
|  | Diabetes | 1220 Diabetes  1222 Type 1 diabetes  1223 Type 2 diabetes  1276 Diabetic eye disease  1468 Diabetic neuropathy/ulcers  1607 Diabetic nephropathy | E10 Insulin-dependent diabetes mellitus  E11 Non-insulin-dependent diabetes mellitus  E13 Other specified diabetes mellitus  E14 Unspecified diabetes mellitus  O24.4 Gestational diabetes |
|  | Diverticular disease | 1458 Diverticular disease/diverticulitis | K57 Diverticular disease of intestine |
|  | Dyspepsia/ulcer | 1138 Gastro-oesophageal reflux/gastric reflux  1139 Oesophagitis/barretts oesophagus  1142 Gastric/stomach ulcers  1143 Gastritis/gastric erosions  1442 Helicobacter pylori  1457 Duodenal ulcer  1474 Hiatus hernia  1510 Dyspepsia/indigestion | K21 Gastro-oesophageal reflux disease  K22 Other diseases of oesophagus  K25 Gastric ulcer  K26 Duodenal ulcer  K27 Peptic ulcer, site unspecified  K28 Gastrojejunal ulcer  K29 Gastritis and duodenitis  K30 Dyspepsia |
|  | Endometriosis | 1402 Endometriosis | N80 Endometriosis |
|  | Epilepsy | 1264 Epilepsy | G40 Epilepsy |
|  | Glaucoma | 1277 Glaucoma | H40 Glaucoma  H42 Glaucoma in diseases classified elsewhere |
|  | Heart failure | 1076 Heart failure/pulmonary oedema  1079 Cardiomyopathy  1588 Hypertrophic cardiomyopathy | I46 Cardiac arrest  I50 Heart failure |
|  | Hepatitis | 1156 Infective/viral hepatitis  1578 hepatitis A  1579 hepatitis B  1580 Hepatitis C  1581 Hepatitis D  1582 Hepatitis E | B15 Acute hepatitis A  B16 Acute hepatitis B  B17 Other acute viral hepatitis  B18 Chronic viral hepatitis  B19 Unspecified viral hepatitis |
|  | Hypertension | 1065 Hypertension  1072 Essential hypertension | I10 Essential (primary) hypertension  I11 Hypertensive heart disease  I12 Hypertensive renal disease  I13 Hypertensive heart and renal disease  I15 Secondary hypertension |
|  | Inflammatory bowel disease (IBD) | 1461 Inflammatory bowel disease  1462 Crohn’s disease  1463 Ulcerative colitis | K50 Crohn disease [regional enteritis]  K51 Ulcerative colitis  K52 Other noninfective gastroenteritis and colitis |
|  | Irritable bowel syndrome (IBS) | 1154 Irritable bowel syndrome | K58 Irritable bowel syndrome |
|  | Liver disease | 1141 Oesophageal varicies  1157 Non-infective hepatitis  1158 Liver failure/cirrhosis  1506 Primary biliary cirrhosis | K70 Alcoholic liver disease  K71 Toxic liver disease  K72 Hepatic failure, not elsewhere classified  K73 Chronic hepatitis, not elsewhere classified  K74 Fibrosis and cirrhosis of liver  K75 Other inflammatory liver diseases  K76 Other diseases of liver  K77 Liver disorders in diseases classified elsewhere |
|  | Méniére’s disease | 1421 Méniére disease | H81.0 Méniére disease |
|  | Migraine | 1265 Migraine | G43 Migraine |
|  | Multiple sclerosis (MS) | 1261 Multiple sclerosis | G35 Multiple sclerosis |
|  | Osteoporosis | 1309 Osteoporosis | M80 Osteoporosis with pathological fracture  M81 Osteoporosis without pathological fracture  M82 Osteoporosis in diseases classified elsewhere |
|  | Painful conditions | 1257 Trapped nerve/compressed nerve  1294 Back problem  1311 Spine arthritis/spondylitis  1312 Prolapsed disc/slipped disc  1313 Ankylosing spondylitis  1436 Headaches (not migraine)  1465 Osteoarthritis  1466 Gout  1476 Sciatica  1478 Cervical spondylosis  1523 Trigeminal neuralgia  1532 Disc problem  1533 Disc degeneration  1534 Back pain  1537 Joint pain  1538 Arthritis  1540 Plantar fasciitis  1541 Carpal tunnel syndrome  1542 Fibromyalgia  1573 Shingles | M45 Ankylosing spondylitis  M46 Other inflammatory spondylopathies  M47 Spondylosis  M48 Other spondylopathies  M49 Spondylopathies in diseases classified elsewhere  M50 Cervical disc disorders  M51 Other intervertebral disc disorders  M53 Other dorsopathies, not elsewhere classified  M54 Dorsalgia  R51 Headache  G44 Other headache syndromes  M10 Gout  M11 Other crystal arthropathies  M12 Other specific arthropathies  M13 Other arthritis  M14 Arthropathies in other diseases classified elsewhere  M72.2 Plantar fasciitis  G50.0 Trigeminal neuralgia  G50.1 Atypical facial pain  G56.0 Carpal tunnel syndrome  M79.7 Fibromyalgia  B02 Herpes zoster (shingles) |
|  | Parkinso‘s disease | 1262 Parkinson’s disease | G20 Parkinson’s disease  G21 Secondary parkinsonism |
|  | Peripheral vascular disease (PVD) | 1067 Peripheral vascular disease  1087 Leg claudication/intermittent claudication | I70.0 Atherosclerosis of aorta  I70.2 Atherosclerosis of arteries of extremities  I70.8 Atherosclerosis of other arteries  I70.9 Generalized and unspecified atherosclerosis  I73.1 Thromboangiitis obliterans [Buerger]  I73.8 Other specified peripheral vascular diseases  I73.9 Peripheral vascular disease, unspecified |
|  | Pernicious anaemia | 1331 Pernicious anaemia | D51.0 Vitamin B12 deficiency anaemia due to intrinsic factor deficiency |
|  | Polycystic ovarian syndrome (PCOS) | 1350 Polycystic ovarian syndrome | E28.2 Polycystic ovarian syndrome |
|  | Prostate conditions (not cancer) | 1207 Prostate problem (not cancer)  1396 Enlarged prostate  1516 Benign prostatic hypertrophy | N40 Hyperplasia of prostate  N41 Inflammatory diseases of pro |
|  | Psoriasis/eczema | 1452 Eczema/dermatitis  1453 Psoriasis | L20 Atopic dermatitis  L21 Seborrhoeic dermatitis  L22 Diaper [napkin] dermatitis  L23 Allergic contact dermatitis  L24 Irritant contact dermatitis  L25 Unspecified contact dermatitis  L26 Exfoliative dermatitis  L27 Dermatitis due to substances taken internally  L28 Lichen simplex chronicus and prurigo  L29 Pruritis  L30 Other and unspecified dermatitis  L40 Psoriasis  L41 Parapsoriasis |
|  | Stroke/transient ischaemic attack (TIA) | 1081 Stroke  1082 Transient ischaemic attack  1086 Subarachnoid haemorrhage  1491 Brain haemorrhage  1583 Ischaemic stroke | I60 Subarachnoid haemorrhage  I61 Intracerebral haemorrhage  I62 Other nontraumatic intracranial haemorrhage  I63 Cerebral infarction  I65 Occlusion and stenosis of precerebral arteries, not resulting in cerebral infarction  I66 Occlusion and stenosis of cerebral arteries, not resulting in cerebral infarction  I67 Other cerebrovascular diseases  I68 Cerebrovascular disorders in diseases classified elsewhere  I69 Sequelae of cerebrovascular disease |
|  | Thyroid conditions | 1224 Thyroid problem (not cancer)  1225 Hyperthyroidism/ thyrotoxicosis  1226 Hypothyroidism/ myxoedema  1428 Thyroiditis  1522 Grave’s disease  1610 Thyroid goitre | E01 Iodine-deficiency-related thyroid disorders and allied conditions  E02 Subclinical iodine-deficiency hypothyroidism  E03 Other hypothyroidism  E04 Other nontoxic goitre  E05 Thyrotoxicosis [hyperthyroidism]  E06 Thyroiditis  E07 Other disorders of thyroid |
| Psychological disorder | Substance use disorder | 1470 anorexia/ bulimia/ other eating disorder | F10 Mental and behavioural disorders due to use of alcohol  F11 Mental and behavioural disorders due to use of opioids  F12 Mental and behavioural disorders due to use of cannabinoids  F13 Mental and behavioural disorders due to use of sedatives or hypnotics  F14 Mental and behavioural disorders due to use of cocaine  F15 Mental and behavioural disorders due to use of other stimulants, including caffeine  F16 Mental and behavioural disorders due to use of hallucinogens  F17 Mental and behavioural disorders due to use of tobacco  F18 Mental and behavioural disorders due to use of volatile solvents  F19 Mental and behavioural disorders due to multiple drug use and use of other psychoactive substances |
|  | Schizophrenia | 1289 schizophrenia | F20 Schizophrenia |
|  | Mood [affective] disorders | 1286 depression  1291 mania/ bipolar disorder/ manic depression  1290 deliberate self-harm/suicide attempt | F30 Manic episode  F31 Bipolar affective disorder  F32 Depressive episode  F33 Recurrent depressive disorder  F34 Persistent mood [affective] disorders  F38 Other mood [affective] disorders  F39 Unspecified mood [affective] disorder |
|  | Neurotic, stress-related and somatoform disorders | 1287 anxiety/panic attacks  1614 stress  1469 post-traumatic stress disorder  1288 nervous breakdown  1615 obsessive compulsive disorder (OCD) | F40 Phobic anxiety disorders  F41 Other anxiety disorders  F42 Obsessive-compulsive disorder  F43 Reaction to severe stress, and adjustment disorders  F44 Dissociative [conversion] disorders  F45 Somatoform disorders  F48 Other neurotic disorders |
| Cognitive disorder | Dementia | 1263 dementia/ Alzheimer/ cognitive impairment | F00 Dementia in Alzheimer's disease  F01 Vascular dementia  F02 Dementia in other diseases classified elsewhere  F03 Unspecified dementia  F05.1 Delirium superimposed on dementia  G30 Alzheimer's disease |
|  | Cognitive Test: Fluid intelligence, Reaction time, Numeric memory, Visuospatial memory, Prospective memory | | |

Note. ICD-10, International Classification of Diseases 10th Revision.

**Table S2. Cognitive tests included in each cognitive domain.^3^**

| **Measurement** | **Cognitive Domain** | **Describe** | **Weight** | **Field ID** |
| --- | --- | --- | --- | --- |
| Fluid intelligence | Verbal and numerical  reasoning | Fluid intelligence score  assessment | The score was an unweighted total of correct responses, from 0 to 13. | 20016 |
| Reaction time | Processing speed | Psychomotor speed was measured by pressing a button as quickly as possible each time a matching pair of symbols was presented onscreen. | The score was the mean time in milliseconds across trials. | 20023 |
| Numeric memory | Working memory | Maximum number of digits remembered correctly | The score was the maximum string length recalled correctly. | 4282 |
| Visuospatial memory | Visual declarative  memory | Number of incorrect  matches made in round | The score was the number of errors made whilst attempting to select the pairs. | 399 |
| Prospective memory | Prospective memory | An instruction was given onscreen asking participants to select a certain shape from an array that would be presented later in the assessment process. When the array later appeared, participants were given up to two chances to select the correct shape. | Performance was scored dichotomously as being correct on the first attempt or not. | 20018 |

**Table S3. Variables for the construction of biological ages**

|  | **Variables in this Study** | **Variable Description** | **Filed ID** |
| --- | --- | --- | --- |
| √ | sbp | Systolic blood pressure, automated reading (mm Hg) | 4080 |
| √ | fev | Forced expiratory volume in 1-second (L) | 3063 |
| √ | totchol | Cholesterol (mmol/L) | 30690 |
| √ | hba1c | Glycated haemoglobin (mmol/mol) | 30750 |
| √ | bun | Urea (mmol/L) | 30670 |
| √* | crp | C-reactive protein (mg/L) | 30710 |
| √* | alp | Alkaline phosphatase (U/L) | 30610 |
| √* | albumin | Albumin (g/L) | 30600 |
| √* | creat | Creatinine (μmol/L) | 30700 |
| * | lymph | Lymphocyte percentage (%) | 30180 |
| * | mcv | Mean sphered cell volume (fL) | 30270 |
| * | glucose | Glucose (mmol/L) | 30740 |
| * | rdw | Red blood cell (erythrocyte) distribution width (%) | 30070 |
| * | wbc | White blood cell (leukocyte) count (10^9 cells/L) | 30000 |

Note. √ indicates indicators used to construct KMD-BA, and * denotes indicators used to construct Phenoage.

**Table S4. Variables used to calculate healthy diet score in the UK biobank^4^**

| **Diet frequency questions** | **Diet component** | **Filed ID** | **Health diet*** |
| --- | --- | --- | --- |
| "On average how many heaped tablespoons of COOKED vegetables would you eat per DAY? (Do not include potatoes; put '0' if you do not eat any)" | Vegetables | 1289 | ≥4 tablespoons/day |
| "About how many pieces of FRESH fruit would you eat per DAY? (Count one apple, one banana, 10 grapes etc as one piece; put '0' if you do not eat any)" | Fresh fruits | 1309 | ≥ 3 pieces/day |
| "How often do you eat oily fish? (e.g. sardines, salmon, mackerel, herring)" | Fish | 1329 | ≥2 times a week. |
| "How often do you eat processed meats (such as bacon, ham, sausages, meat pies, kebabs, burgers, chicken nuggets)?" | Processed meat | 1349 | < 2 times a week |
| "How often do you eat beef? (Do not count processed meats)" | Unprocessed red meat | 1369 | <2 times a week. |
| "How often do you eat lamb/mutton? (Do not count processed meats)" |  | 1379 | <2 times a week |
| "How often do you eat pork? (Do not count processed meats such as bacon or ham)" |  | 1389 | <2 times a week |

Note. # A score of 1 is assigned for unprocessed red meat if at least one criterion of a healthy diet is met; if none are met, a score of 0 is given. Scores for each category, based on meeting healthy diet criteria, are summed for a total score ranging from 0 to 5.

**Table S5. Association between accelerated aging and multimorbidity involving physical, psychological, and cognitive disorders at baseline**

|  | **Single Disorder (N=183,505)** | | | **Dual Comorbidity (N=77,764)** | | | **Triple Comorbidity (N=10,696)** | | |  |
| --- | --- | --- | --- | --- | --- | --- | --- | --- | --- | --- |
|  | **N_case_ /N_total_** | **OR (95%CI)** | **P** | **N_case_ /N_total_** | **OR (95%CI)** | **P** | **N_case_ /N_total_** | **OR (95%CI)** | **P** |  |
| **KDM-BA acceleration (Per-SD increase)** | | | | |  |  |  |  |  |  |
|  | 57,525/ 105,059 | 1.03 (1.01-1.05) | <0.001 | 29,383/ 105,059 | 1.07 (1.05-1.10) | <0.001 | 4,490/ 105,059 | 1.20 (1.15-1.26) | <0.001 |  |
| **KDM-BA acceleration (Quartiles)** | | | |  |  |  |  |  |  |  |
| Q1 | 46,020 /83,003 | Ref | | 15,939 /83,003 | Ref | | 1,995 /83,003 | Ref | |  |
| Q2 | 46,174 /83,003 | 1.12 (1.09-1.15) | <0.001 | 18,004 /83,003 | 1.16 (1.12-1.20) | <0.001 | 2,306 /83,003 | 1.13 (1.05-1.21) | 0.001 |  |
| Q3 | 45,990 /83,003 | 1.22 (1.19-1.25) | <0.001 | 19,976 /83,003 | 1.34 (1.30-1.39) | <0.001 | 2,683 /83,003 | 1.35 (1.26-1.45) | <0.001 |  |
| Q4 | 45,321 /83,003 | 1.59 (1.54-1.64) | <0.001 | 23,845 /83,003 | 1.93 (1.86-2.00) | <0.001 | 3,712 /83,003 | 2.18 (2.03-2.34) | <0.001 |  |
| *P* for trend | <0.001 | | | <0.001 | | | <0.001 | | |  |
| **PhenoAge acceleration (Per-SD increase)** | | | |  |  |  |  |  |  |  |
|  | | 15,016 / 28,833 | 1.01 (0.99-1.03) | 0.205 | 9,606 /12,249 | 1.04 (1.01-1.06) | <0.001 | 1,731/ 12,249 | 1.12 (1.07-1.17) | <0.001 |
| **PhenoAge acceleration (Quartiles)** | | | | |  |  |  |  |  |  |
| Q1 | | 45,972 /83,003 | Ref | | 15,968 /83,003 | Ref | | 1,875 /83,003 | Ref | |
| Q2 | | 46,226 /83,003 | 1.12 (1.09-1.14) | <0.001 | 17,865 /83,003 | 1.19 (1.16-1.23) | <0.001 | 2,271 /83,003 | 1.29 (1.21-1.38) | <0.001 |
| Q3 | | 46,410 /83,003 | 1.26 (1.23-1.29) | <0.001 | 19,676 /83,003 | 1.41 (1.37-1.47) | <0.001 | 2,679 /83,003 | 1.62 (1.51-1.73) | <0.001 |
| Q4 | | 44,897 /83,003 | 1.61 (1.56-1.66) | <0.001 | 24,255 /83,003 | 2.07 (2.00-2.14) | <0.001 | 3,871 /83,003 | 2.69 (2.51-2.88) | <0.001 |
| *P* for trend | | <0.001 | | | <0.001 | | | <0.001 | | |

Note. The table presents the odds ratios (ORs) for each condition, along with their corresponding 95% confidence intervals (CIs) and p-values.

The model has been adjusted for various factors to account for potential confounding variables, such as age, sex, ethnicity, BMI, smoking status, alcohol intake, healthy diet score, physical activity, Townsend deprivation index, employment status, income, and air pollution. The missing data for covariates were handled via multiple imputation.

**Table S6. Association between accelerated aging at base line with incident multimorbidity involving physical, psychological, and cognitive disorders at follow-up (fully adjusted model)**

|  | **Single Disorder (N= 31,713)** | | | **Dual Disorders& Triple Disorders (N=** **4,187)** | | |  |
| --- | --- | --- | --- | --- | --- | --- | --- |
|  | **N_case_ /N_total_** | **HR (95%CI)** | **P** | **N_case_ /N_total_** | **HR (95%CI)** | **P** |  |
| **KDM-BA acceleration** | | | | |  |  |  |
|  | 7,685 /13,611 | 1.12 (1.09-1.15) | <0.001 | 1,220 /13,611 | 1.32 (1.23-1.41) | <0.001 |  |
| **KDM-BA acceleration (Quartiles)** | | | |  |  |  |  |
| Q1 | 7,731 /15,012 | Ref | | 890 /15,012 | Ref | |  |
| Q2 | 7,708 /15,012 | 1.04 (1.01- 1.08) | 0.009 | 909 /15,012 | 1.04 (0.95-1.14) | 0.434 |  |
| Q3 | 7,870 /15,012 | 1.07 (1.04- 1.11) | <0.001 | 1,062 /15,012 | 1.19 (1.09-1.30) | <0.001 |  |
| Q4 | 8,404 /15,011 | 1.16 (1.13- 1.20) | <0.001 | 1,326 /15,011 | 1.44 (1.32-1.57) | <0.001 |  |
| **PhenoAge acceleration** | | | |  |  |  |  |
|  | | 1,475 /2,480 | 1.19 (1.13-1.26) | <0.001 | 308/ 2,480 | 1.49 (1.32-1.68) | <0.001 |
| **PhenoAge acceleration (Quartiles)** | | | | |  |  |  |
| Q1 | | 7,640 /15,012 | Ref | | 868 /15,014 | Ref | |
| Q2 | | 7,691 /15,012 | 1.02 (0.99- 1.06) | 0.140 | 828 /15,013 | 0.90 (0.81-0.99) | 0.025 |
| Q3 | | 7,909 /15,012 | 1.07 (1.03- 1.11) | <0.001 | 1,071 /15,013 | 1.14 (1.04-1.24) | 0.005 |
| Q4 | | 8,473 /15,011 | 1.17 (1.14- 1.21) | <0.001 | 1,420 /15,013 | 1.41 (1.30-1.54) | <0.001 |

Note. Among the 600,474 participants included in the baseline analysis, the number of individuals who developed somatic-cognitive-mental triple comorbidity during follow-up was relatively low (n = 112). To ensure the robustness of the results, both dual and triple comorbidity were combined into a single group for analysis. The model was adjusted for multiple potential confounding variables, including age, sex, ethnicity, BMI, smoking status, alcohol intake, healthy diet score, physical activity, the Townsend deprivation index, household income, and air pollution. Missing data for covariates were addressed using multiple imputation.

| **Table S7. Characteristics of study participants according to health status** | | | | |  |  |
| --- | --- | --- | --- | --- | --- | --- |
|  | **Total**  **Life expectancy (95%)** | **No Condition**  **Life expectancy (95%)** | **Single Disorder**  **Life expectancy (95%)** | **Dual Comorbidity**  **Life expectancy (95%)** | | **Triple Comorbidity**  **Life expectancy (95%)** |
|  |  |  |  |  |  |  |
| At 45 years | 40.5 (39.9,41.0) | 42.9 (41.1, 44.7) | 40.5 (39.7, 41.3) | 39.0 (38.0, 39.9) | | 37.6 (35.5, 39.8) |
| At 55 years | 30.8 (30.2,31.3) | 33.2 (31.4, 35.0) | 30.7 (30.0, 31.5) | 29.1 (28.2, 23.0) | | 28.1 (26.1, 30.1) |
| At 65 years | 21.4 (20.9,21.9) | 23.8 (22.1, 25.5) | 21.4 (20.7, 22.1) | 19.8 (18.9, 20.6) | | 19.1 (17.2, 21.0) |

Note. Models were adjusted for age, sex, ethnicity, BMI, smoking status, alcohol intake, healthy diet score, physical activity, Townsend deprivation index, household income, and air pollution.

**Table S8. Association between accelerated aging and multimorbidity involving physical, psychological, and cognitive disorders at baseline with complete covariates (n=201,564)**

|  | **Single Disorder(N=112,562)** | | | | **Dual Comorbidity (N=44,092)** | | | | **Triple Comorbidity (N=5,401)** | | |  |
| --- | --- | --- | --- | --- | --- | --- | --- | --- | --- | --- | --- | --- |
|  | **N_case_ /N_total_** | **OR (95%CI)** | **P** |  | **N_case_ /N_total_** | **OR (95%CI)** | **P** |  | **N_case_ /N_total_** | **OR (95%CI)** | **P** |  |
| **KDM-BA acceleration (Per-SD increase)** | | | | | |  |  | |  |  |  |  |
|  | 32,851 / 58,457 | 1.06 (1.02-1.07) | <0.001 | | 15,196 /58,457 | 1.06 (1.03-1.10) | <0.001 | | 2,072 /58,457 | 1.18 (1.11-1.26) | <0.001 |  |
| **KDM-BA acceleration (Quartiles)** | | | | |  |  |  | |  |  |  |  |
| Q1 | 27,975 /50,391 | Ref | | | 9,221 /50,391 | Ref | | | 1,013 /50,391 | Ref | |  |
| Q2 | 28,093 /50,391 | 1.10 (1.07-1.14) | <0.001 | | 10,274 /50,391 | 1.13 (1.08-1.18) | <0.001 | | 1,143 /50,391 | 1.10 (1.00-1.22) | 0.042 |  |
| Q3 | 28,087 /50,391 | 1.20 (1.16-1.25) | <0.001 | | 11,340 /50,391 | 1.30 (1.24-1.35) | <0.001 | | 1,412 /50,391 | 1.40 (1.28-1.55) | <0.001 |  |
| Q4 | 28,407 /50,391 | 1.58 (1.52-1.65) | <0.001 | | 13,257 /50,391 | 1.76 (1.68-1.85) | <0.001 | | 1,833 /50,391 | 2.02 (1.83-2.22) | <0.001 |  |
| *P* for trend | <0.001 | | | | <0.001 | | | | <0.001 | | |  |
| **PhenoAge acceleration (Per-SD increase)** | | | | |  |  |  | |  |  |  |  |
|  | | 8,353 /15,460 | 1.01 (0.99 -1.04) | 0.348 | | 4,796 /15,460 | 1.02 (9.89-1.05) | 0.226 | | 755 /15,460 | 1.12 (1.05-1.20) | <0.001 |
| **PhenoAge acceleration (Quartiles)** | | | | | |  |  |  | |  |  |  |
| Q1 | | 28,011 /50,391 | Ref | | | 9,088 /50,391 | Ref | | | 977 /50,391 | Ref | |
| Q2 | | 28,152 /50,391 | 1.10 (1.07-1.14) | <0.001 | | 10,161 /50391 | 1.17 (1.13-1.22) | <0.001 | | 1,165 /50,391 | 1.25 (1.14-1.37) | <0.001 |
| Q3 | | 28,382 /50,391 | 1.20 (1.16 -1.25) | <0.001 | | 11,308 /50,391 | 1.42 (1.36-1.48) | <0.001 | | 1,323 /50,391 | 1.53 (1.40-1.68 | <0.001 |
| Q4 | | 28,017 /50,391 | 1.58 (1.52-1.65) | <0.001 | | 13,535 /50,391 | 1.92 (1.84-2.01) | <0.001 | | 1,936 /50,391 | 2.42 (2.21-2.65) | <0.001 |
| *P* for trend | | <0.001 | | | | <0.001 | | | | <0.001 | | |

The models have been adjusted for age, sex, ethnicity, BMI, smoking status, alcohol intake, healthy diet score, physical activity, Townsend deprivation index, household income, and air pollution.

**Table S9. Association between baseline accelerated aging and incident of multimorbidity involving physical, psychological, and cognitive disorders at follow-up with complete covariates(n=39,509)**

|  | **Single Disorder**  **(N= 19,109)** | | | | **Dual Comorbidity & Triple Comorbidity**  **(N=** **2,609)** | | | |  |
| --- | --- | --- | --- | --- | --- | --- | --- | --- | --- |
|  | **N_case_ /N_total_** | **HR (95%CI)** | **P** |  | **N_case_ /N_total_** | **HR (95%CI)** | **P** |  |  |
| **KDMAge acceleration** | | | | | |  |  | |  |
|  | 4,560 / 8,338 | 1.11 (1.07 -1.15) | <0.001 | | 707 /8,338 | 1.32 (1.21 - 1.44) | <0.001 | |  |
| **KDM-BA acceleration (Quartiles)** | | | | |  |  |  | |  |
| Q1 | 4,986 /9,878 | Ref | | | 537 /9,878 | Ref | | |  |
| Q2 | 5,005 /9,877 | 1.05 (1.01 - 1.10) | 0.010 | | 584 /9,877 | 1.12 (1.00 – 1.26) | 0.056 | |  |
| Q3 | 5,050 /9,877 | 1.08 (1.04 - 1.12) | <0.001 | | 657 /9,877 | 1.26 (1.12 - 1.42) | <0.001 | |  |
| Q4 | 5,359 /9,877 | 1.15 (1.11 - 1.20) | <0.001 | | 831 /9,877 | 1.52 (1.36 - 1.71) | <0.001 | |  |
| **PhenoAge acceleration** | | | | |  |  |  | |  |
|  | | 865 /1,502 | 1.18 (1.10 - 1.26) | <0.001 | | 168 /1,502 | 1.42 (1.21-1.67) | <0.001 | |
| **PhenoAge acceleration (Quartiles)** | | | | | |  |  |  | |
| Q1 | | 4,968 /9,878 | Ref | | | 528 /9,878 | Ref | | |
| Q2 | | 4,924 /9,877 | 1.00 (0.96 - 1.04) | 0.908 | | 544 /9,877 | 0.98 (0.87 -1.10) | 0.713 | |
| Q3 | | 5,097 /9,877 | 1.06 (1.02 - 1.11) | 0.002 | | 671 /9,877 | 1.18 (1.05 -1.32) | 0.005 | |
| Q4 | | 5,411 /9,877 | 1.15 (1.10 - 1.20) | <0.001 | | 866 /9,877 | 1.46 (1.30 -1.63) | <0.001 | |

Note. The models have been adjusted for age, sex, ethnicity, BMI, smoking status, alcohol intake, healthy diet score, physical activity, Townsend deprivation index, household income, and air pollution.

**Table S10. Association of accelerated aging with follow-up multimorbidity excluding baseline and early-onset disorders (n=48,235*)**

|  | **Single Disorder**  **(N= 19,938)** | | | | **Dual Comorbidity & Triple Comorbidity**  **(N=** **3,387)** | | | |  |
| --- | --- | --- | --- | --- | --- | --- | --- | --- | --- |
|  | **N_case_ /N_total_** | **HR (95%CI)** | **P** |  | **N_case_ /N_total_** | **HR (95%CI)** | **P** |  |  |
| **KDMAge acceleration** | | | | | |  |  | |  |
|  | 4,685 /10,649 | 1.10 (1.06 - 1.13) | <0.001 | | 952 /10,649 | 1.32 (1.22 - 1.43) | <0.001 | |  |
| **KDM-BA acceleration (Quartiles)** | | | | |  |  |  | |  |
| Q1 | 4,907 /12,059 | Ref | | | 718 /12,059 | Ref | | |  |
| Q2 | 4,841 /12,059 | 1.03 (0.99 – 1.07) | 0.124 | | 738 /12,059 | 1.04 (0.94 - 1.16) | 0.432 | |  |
| Q3 | 4,934 /12,059 | 1.06 (1.02 - 1.10) | 0.006 | | 871 /12,059 | 1.23 (1.11 - 1.36) | <0.001 | |  |
| Q4 | 5,256 /12,058 | 1.13 (1.08 - 1.17) | <0.001 | | 1,060 /12,058 | 1.45 (1.31 - 1.60) | <0.001 | |  |
| **PhenoAge acceleration** | | | | |  |  |  | |  |
|  | | 914 /1,915 | 1.04 (1.02 - 1.07) | <0.001 | | 243 /1,915 | 1.53 (1.34-1.75) | <0.001 | |
| **PhenoAge acceleration (Quartiles)** | | | | | |  |  |  | |
| Q1 | | 4,823 /12,059 | Ref | | | 714 /12,059 | Ref | | |
| Q2 | | 4,818 /12,059 | 1.02 (0.97 - 1.09) | 0.336 | | 658 /12,059 | 0.87 (0.78 -0.96) | 0.008 | |
| Q3 | | 4,921 /12,059 | 1.02 (0.97 - 1.08) | 0.420 | | 866 /12,059 | 1.13 (1.02 -1.24) | 0.019 | |
| Q4 | | 5,376 /12,058 | 1.15 (1.08 - 1.21) | <0.001 | | 1,149 /12,058 | 1.38 (1.26 -1.52) | <0.001 | |

Note. * Excluding individuals with disorders at baseline and those who developed disorders or died within the first 5 years of follow-up.

The models have been adjusted for age, sex, ethnicity, BMI, smoking status, alcohol intake, healthy diet score, physical activity, Townsend deprivation index, household income, and air pollution.

**Table S11. Association between telomere length and multimorbidity at baseline.**

|  | **Single Disorder(N=177,042)** | | |  | **Dual Comorbidity(N=75,022)** | | |  | **Triple Comorbidity(N=10,258)** | | |
| --- | --- | --- | --- | --- | --- | --- | --- | --- | --- | --- | --- |
|  | **N_case_/N_total_** | **OR (95%CI)** | **P** |  | **N_case_/N_total_** | **OR (95%CI)** | **P** |  | **N_case_/N_total_** | **OR (95%CI)** | **P** |
| **Z-adjusted T/S log(Quartiles)** | | | | |  |  |  | |  |  |  |
| Q1 | 44,386 /80,057 | Ref | | | 20,126 /80,057 | Ref | | | 2,847 /80,057 | Ref | |
| Q2 | 44,488 /80,057 | 0.99 (0.96-1.01) | 0.337 | | 18,855 /80,057 | 0.95 (0.92-0.98) | 0.002 | | 2,630 /80,057 | 0.96 (0.90-1.02) | 0.168 |
| Q3 | 44,181 /80,057 | 0.98 (0.96-1.01) | 0.262 | | 18,329 /80,057 | 0.94 (0.91-0.97) | <0.001 | | 2,519 /80,057 | 0.94 (0.88-1.00) | 0.067 |
| Q4 | 43,987 /80,056 | 0.98 (0.95-1.01) | 0.236 | | 17,712 /80,056 | 0.91 (0.88-0.94) | <0.001 | | 2,262 /80,056 | 0.86 (0.81-0.93) | <0.001 |
| *P* for trend | 0.300 | | | | <0.001 | | | | <0.001 | | |

The models have been adjusted for age, sex, ethnicity, BMI, smoking status, alcohol intake, healthy diet score, physical activity, Townsend deprivation index, household income, and air pollution. The missing data for covariates were handled using multiple imputation.

*Telomere length (TL) in peripheral blood leukocytes was assessed using the multiplex quantitative polymerase chain reaction (qPCR) method by researchers at the University of Leicester, England. TL was quantified as the ratio of the number of TL repeats (T) to a reference single-copy gene (S), resulting in a T/S ratio. Both T and S measurements were derived from either a calibrator sample composed of pooled DNA from 20 individuals or from a corresponding standard curve. To address the skewed distribution of T/S ratios and variations arising from different quantification methods, a natural logarithm-transformed z-standardized T/S ratio (Data-Field 22192) was calculated based on the distribution of all participants who underwent TL measurement, and this ratio was utilized in the current study.

**Table S12. Association between baseline telomere length and incident single disorder, comorbidity and death during follow-up.(n=57,905)**

|  | **Single Disorder (31,713)** | | |  | **Comorbidity (4,187)** | | | |  | **Death (2,485)** | | |  |
| --- | --- | --- | --- | --- | --- | --- | --- | --- | --- | --- | --- | --- | --- |
|  | **N_case_/N_total_** | **HR (95%CI)** | **P** |  | **N_case_/N_total_** | **HR (95%CI)** | | **P** |  | **N_case_/N_total_** | **HR (95%CI)** | **P** |  |
| Z-adjusted T/S log ^*^(Quartiles) | | | | | |  |  | |  | |  |  |  |
| Q1 | | 6,343 /14,477 | Ref | | | 1,177 /14,477 | Ref |  | | | 801 /14,477 | Ref |  |
| Q2 | | 6,812 /14,476 | 0.95 (0.92-0.98) | 0.003 | | 1,035 /14,476 | 0.91 (0.83-0.99) | | 0.021 | | 610 /14,476 | 0.90 (0.81-1.00) | 0.041 |
| Q3 | | 6,988 /14,476 | 0.98 (0.95-1.01) | 0.165 | | 919 /14,476 | 0.86 (0.79-0.94) | | 0.001 | | 520 /14,476 | 0.87 (0.77-0.97) | 0.011 |
| Q4 | | 7,163 /14,476 | 0.98 (0.95-1.01) | 0.270 | | 918 /14,476 | 0.89 (0.82-0.97) | | 0.010 | | 461 /14,476 | 0.88 (0.78-0.99) | 0.033 |

The models have been adjusted for age, sex, ethnicity, BMI, smoking status, alcohol intake, healthy diet score, physical activity, Townsend deprivation index, household income, and air pollution. The missing data for covariates were handled using multiple imputation.

*Telomere length (TL) in peripheral blood leukocytes was assessed using the multiplex quantitative polymerase chain reaction (qPCR) method by researchers at the University of Leicester, England. TL was quantified as the ratio of the number of TL repeats (T) to a reference single-copy gene (S), resulting in a T/S ratio. Both T and S measurements were derived from either a calibrator sample composed of pooled DNA from 20 individuals or from a corresponding standard curve. To address the skewed distribution of T/S ratios and variations arising from different quantification methods, a natural logarithm-transformed z-standardized T/S ratio (Data-Field 22192) was calculated based on the distribution of all participants who underwent TL measurement, and this ratio was utilized in the current study.

**Reference**

1 Klemera, P. & Doubal, S. A new approach to the concept and computation of biological age. *Mech Ageing Dev* **127**, 240-248 (2006). <https://doi.org/10.1016/j.mad.2005.10.004>

2 Zhou, Y. *et al.* Associations between socioeconomic inequalities and progression to psychological and cognitive multimorbidities after onset of a physical condition: a multicohort study. *EClinicalMedicine* **74**, 102739 (2024). <https://doi.org/10.1016/j.eclinm.2024.102739>

3 Cullen, B., Smith, D. J., Deary, I. J., Evans, J. J. & Pell, J. P. The 'cognitive footprint' of psychiatric and neurological conditions: cross-sectional study in the UK Biobank cohort. *Acta Psychiatr Scand* **135**, 593-605 (2017). <https://doi.org/10.1111/acps.12733>

4 Cui, F. *et al.* Early-life exposure to tobacco, genetic susceptibility, and accelerated biological aging in adulthood. *Sci Adv* **10**, eadl3747 (2024). <https://doi.org/10.1126/sciadv.adl3747>
